# Molecular Basis for the Remarkably Different Gas-Phase Behavior of Deprotonated Thyroid Hormones Triiodothyronine (T3) and Reverse Triiodothyronine (rT3): A Clue for Their Discrimination?

**DOI:** 10.1021/acs.analchem.1c03892

**Published:** 2021-10-29

**Authors:** Davide Corinti, Barbara Chiavarino, Mattia Spano, Aura Tintaru, Simonetta Fornarini, Maria Elisa Crestoni

**Affiliations:** † Dipartimento di Chimica e Tecnologie del Farmaco, Università di Roma “La Sapienza”, Roma I-00185, Italy; ‡ Aix Marseille Univ, CNRS, Institut de Chimie Radicalaire, UMR 7273, Marseille 13397, France

## Abstract

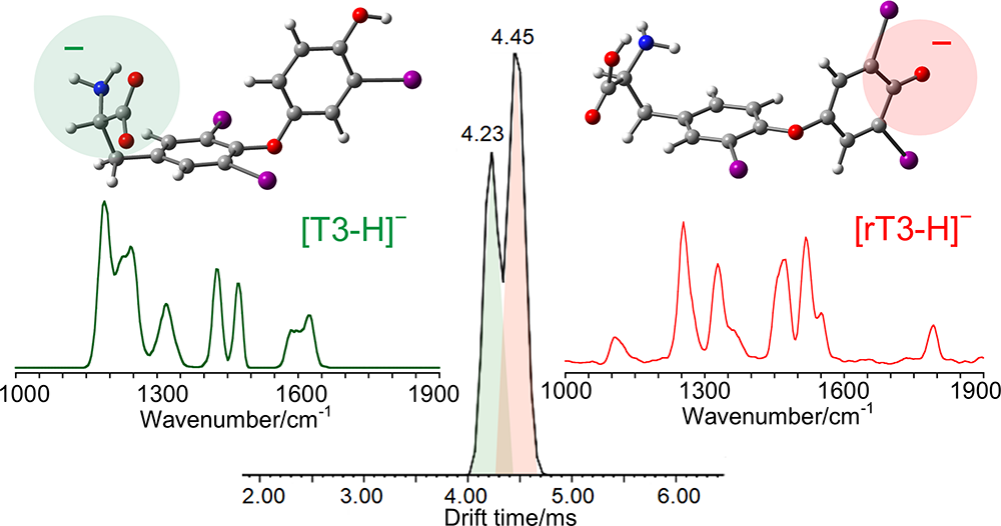

Thyroid hormones are biologically active small molecules responsible for growth and development regulation, basal metabolic rate, and lipid and carbohydrate metabolism. Liquid chromatography mass spectrometry (LC–MS) can be used to quantify thyroid hormones blood level with high speed and selectivity, aiming to improve the diagnosis and treatment of the severe pathological conditions in which they are implicated, i.e., hypo- and hyperthyroidism. In this work, the gas-phase behavior of the isomeric thyroid hormones triiodothyronine (T3) and reverse triiodothyronine (rT3) in their deprotonated form was studied at a molecular level using MS-based techniques. Previously reported collision-induced dissociation experiments yielded distinct spectra despite the high structural similarity of the two compounds, suggesting different charge sites to be responsible. Infrared multiple photon dissociation spectroscopy on [T3-H]^−^ and [rT3-H]^−^ was performed, and the results were interpreted using DFT and MP2 calculations, assessing the prevalence of T3 in the carboxylate form and rT3 as a phenolate isomer. The different deprotonation sites of the two isomers were also found to drive their ion-mobility behavior. In fact, [T3-H]^−^ and [rT3-H]^−^ were successfully separated. Drift times were correlated with collisional cross section values of 209 and 215 Å^2^ for [T3-H]^−^ and [rT3-H]^−^, respectively. Calculations suggested the charge site to be the main parameter involved in the different mobilities of the two anions. Finally, bare [T3-H]^−^ and [rT3-H]^−^ were made to react with neutral acetylacetone and trifluoroacetic acid, confirming rT3 to be more acidic than T3 in agreement with the calculated gas-phase acidities of T3 and rT3 equal to 1345 and 1326 kJ mol^–1^, respectively.

## Introduction

**Scheme 1:**
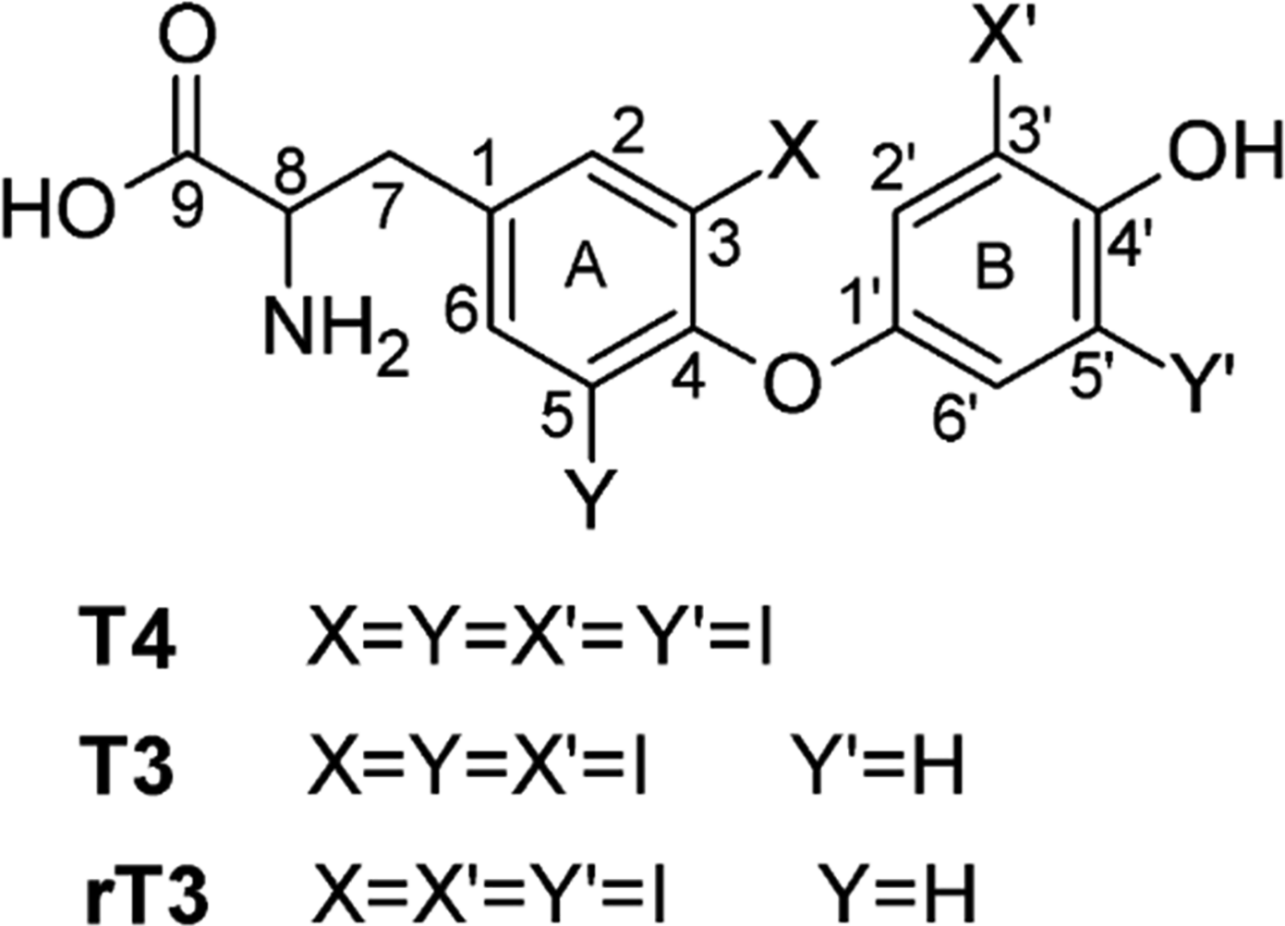
Schematic Representation of Tetraiodothyronine (T4), Triiodothyronine (T3), and Reverse Triiodothyronine (rT3)

Triiodothyronine (3,5,3′-triiodothyronine, T3) and reverse triiodothyronine (3,3′,5′-triiodothyronine, rT3) are thyroid hormones mostly produced by deiodination of thyroxine (3,5,3′,5′-tetraiodothyronine, T4). T4 represents the most abundant thyroid hormone in the bloodstream followed by T3 and finally by rT3. T4 is synthetized in the thyroid gland by oxidative coupling of two molecules of the amino acid tyrosine appropriately iodinated in the 3 and 5 positions of the phenolic ring.^1^ Thyroid hormones are the sole endogenous molecules containing iodine. Their structures are reported in Scheme 1. Thyroid hormones are responsible for the regulation of growth and development, basal metabolic rate, and lipid and carbohydrate metabolism.^2−4^ T4 and T3 share similar activities, but the latter is recognized to be more active. On the contrary, rT3 is reported to have a role as a T3 competitive inhibitor.^5^ Moreover, there is evidence of an inhibiting activity of rT3 on deiodinase, which is responsible for the production of T3 from T4.^5,6^ Thyroid hormones are involved in two pathological conditions, hyper- and hypothyroidism. Hyperthyroidism is characterized by high blood levels of T3 and low ones of thyroid-stimulating hormone (TSH). If not treated, then hyperthyroidism can lead to neurological disorders, osteoporosis, excessive weight loss, palpitations, and eventually heart failure.^7−9^ On the other hand, low blood levels of thyroid hormones and excessive ones of TSH are characteristic of hypothyroidism, which in newborns and children can impair growth and neurological development, while in adults it is connected to obesity and lack of energy.^9−11^

Monitoring the blood levels of TSH, T3, and T4 is crucial to diagnose thyroid diseases and to propose a successful therapeutic plan.^8−11^ TSH is considered the most sensitive indicator of hyper- and hypothyroidism; however, its titration has to be accompanied by the measuring of T4 and T3 blood levels in order to evaluate the severity of the disease.^8^ Accurate measurement of hormone levels is thus needed, also considering that subclinical hyperthyroidism can lead to chronic damage of heart and blood vessels.^12,13^

In clinical practice, electro-chemiluminescent immunoassay is currently used to sample thyroid hormones.^14^ This method is time consuming and lacks sensitivity, thus impairing the diagnosis and management of the two medical conditions, in particular of hypothyroidism. In fact, in that case, the hormones level can be too low to be appropriately quantified.^14−17^ Sensitivity is even lower when samples are analyzed with enzyme-linked immunosorbent assay for the quantification of total T3 and T4, which is a common practice in laboratories with scarcity of specialized instrumentations.^17−20^ Alternative techniques are therefore desirable to improve diagnosis and treatment of thyroid dysfunctions.

Mass spectrometry coupled with liquid chromatography (LC–MS) can act as a game changer for thyroid hormones and TSH analyses, allowing to simultaneously analyze and quantify all the species of interest with high sensitivity and selectivity and also discriminating free hormones from protein-bound ones.^18,21−27^ One of the main issues is, however, to differentiate T3 from rT3 considering that they are isomeric molecules, which present a largely similar structure. Eventually, it has been found that the behavior of deprotonated ions [T3-H]^−^ and [rT3-H]^−^ when submitted to collision-induced dissociation (CID) is rather different, thus permitting to separately quantify the two isomers.^28,29^ Blanksby et al. observed that the CID of [T3-H]^−^ follows the same fragmentation paths as the corresponding deprotonated ion of T2 (3,5-diiodothyronine), suggesting that the additional iodine on the outer ring does not modify the unimolecular chemistry of the anion. On the other hand, [rT3-H]^−^ shows significantly different fragments in agreement with the possibility that the increased acidity of the phenolic moiety, due to the presence of the 3′,5′-diiodination, could lead to the formation of phenoxide isomers,^29^ thus suggesting that the characteristic fragmentation spectra of [T3-H]^−^ and [rT3-H]^−^ could be related to different deprotonation sites in the two isomers.

In order to gain insights on this assumption, this work reports on a study whereby [T3-H]^−^ and [rT3-H]^−^, produced by electrospray ionization (ESI) and mass selected, were submitted to IR multiple photon dissociation (IRMPD) spectroscopy, an approach that permits obtaining of the IR features of the ions in the gas phase.^30−32^ IRMPD spectroscopy, coupled with quantum chemical calculations of the IR spectra of candidate isomers and conformers, has in fact proven its efficacy in releasing structural information on several anionic species, such as deprotonated natural and phosphorylated amino acids,^33,34^ tyrosine and 3-nitrotyrosine complexed with halide ions,^35^ and deprotonated pantothenic acid.^36^ Several studies have also assessed that isomeric forms are retained from the solution to the gas phase, given the soft ionization conditions that ESI permits,^37−40^ thus confirming IRMPD spectroscopy as a powerful and viable technique to assess the deprotonation sites of anions generated in solution.^39,40^ Spectroscopic data are compared with results of ion-molecule reactions (IMR) in the cell of a Fourier transform ion cyclotron resonance (FT-ICR) mass spectrometer. In particular, deprotonated T3 and rT3 were allowed to react with volatile acids and diethylmethoxyborane, which was shown to react selectively with phenoxide anions.^40−42^ Finally, this work also reports on ion mobility mass spectrometry (IM-MS) analyses of [T3-H]^−^ and [rT3-H]^−^ with the aim to correlate the experimentally obtained collision cross section (CCS) values to the ones calculated for distinct deprotonated isomers.^43−45^ On a side note, the different IM behavior of [T3-H]^−^ and [rT3-H]^−^ could eventually be exploited to build novel analytical methods for the detection and quantification of T3 and rT3.^46,47^

## Materials and Methods

### Sample Preparation

Triiodothyronine (3,5,3′-triiodothyronine, T3) and reverse triiodothyronine (3,3′,5′-triiodothyronine, rT3) were bought from Sigma-Aldrich, Milan, IT and used without further purification. T3 was dissolved in methanol of MS-grade to a final concentration of 10^–5^ M with the addition of ammonia (0.1% v/v). rT3 is provided as a methanolic solution already basified with ammonia, which was diluted in methanol to reach the same concentration as T3, i.e., 10^–5^ M. Acetylacetone, trifluoroacetic acid (TFA), and diethylmethoxyborane (DEMB) were commercial products obtained from Sigma-Aldrich to be used as neutral reagents for ion–molecule reactions.

### Tandem MS and IM Analysis

Traveling wave ion mobility mass spectrometry (TWIMS-MS) experiments were performed with a Synapt G2 HDMS quadrupole/time-of-flight mass spectrometer (Waters, Manchester, UK) equipped with an ESI source operated in negative mode for the present experiments. Samples were introduced at a 10 μL/min flow rate (capillary voltage: 2.27 kV, sampling cone voltage: 50 V) under a curtain gas (N_2_) flow of 100 L/h at 35 ° C. Accurate mass experiments were performed using reference ions from the CH_3_COONa external standard *via* a LockSpray interface. All ESI-MS/MS spectra were recorded in the 50–1500 *m*/*z* range, with a trap bias DC voltage of 45 V, a helium cell gas flow of 180 mL/min, and a trap collision energy of 30 eV. For ESI-IM-MS/MS spectra, a transfer collision energy of 30 eV was used. All data analyses were carried out using the MassLynx4.1 and DriftScope 2.1 programs provided by Waters. Drift times were correlated to the CCSs using polyalanine oligomers to calibrate the mobility data.^48^

### IRMPD Analysis

IRMPD spectra in the 800–1800 cm^–1^ frequency range were recorded at the free-electron laser (FEL) beamline of the Centre Laser Infrarouge d’Orsay (CLIO).^49^ The FEL beamline (operated at 42.3 MeV for the present experiments) is coupled with a hybrid FT-ICR tandem mass spectrometer (APEX-Qe Bruker Daltonics)^50^ equipped with a 7.0 T actively shielded magnet and a quadrupole–hexapole interface. The ions of interest were generated by direct injection of the T3 and rT3 solutions into the ESI source of the mass spectrometer, mass isolated in the quadrupole sector, and accumulated in the hexapole for 1 ms before being irradiated in the FT-ICR cell with the IR FEL light operating at a repetition rate of 25 Hz. Irradiation times were 220 and 300 ms for [T3-H]^−^ and [rT3-H]^−^, respectively. The photofragmentation products were mass analyzed, and IRMPD spectra were obtained by plotting the photofragmentation yield *R* (corresponding to −log[*I*_p_/(*I*_p_ + Σ*I*_f_)], where *I*_p_ and Σ*I*_f_ are the integrated abundances of the precursor ion and the sum of the fragments, respectively) as a function of the photon energy.^51^

### Ion-Molecule Reactions

High-resolution mass analyses (resolving power FWHM at 750 *m*/*z* of ca. 20,000) and ion-molecule reactions were performed in the cell of an FT-ICR mass spectrometer (Bruker BioApex) equipped with an Apollo I ESI source, a 4.7 T magnet, and an infinity cell. Samples were directly injected at a 160 μL h^–1^ flow rate and desolvated by a nitrogen counter-current heated at 400 K. Finally, the mass selected ions were allowed to react with neutral reagents at a stationary pressure in a range of 1.0–10 × 10^–8^ mbar at a nominal FT-ICR cell temperature of 300 K.^52,53^ The pressure was measured with a cold-cathode sensor (IKR Pfeiffer Balzers S.p.A., Milan, Italy) calibrated by using the rate constant *k* = 1.1 × 10^–9^ cm^3^ s^–1^ for the reference reaction CH_4_^+**·**^ + CH_4_ → CH_5_^+^ + CH_3_^**·**^ and corrected for different response factors.^54^ Pseudo-first-order rate constants *k*′, obtained from the semilog plot of the parent ion decay with increasing reaction time, were divided by the neutral concentration to give the second order rate constant (*k*_exp_). For competitive reactions, *k*_exp_ was multiplied by the ratio between the abundance of the reaction product of interest and that of the sum of all products. The reaction efficiency (ϕ) was calculated by dividing *k*_exp_ by the collisional rate constant calculated using the parametrized trajectory theory (*k*_ADO_).^55,56^ The reaction efficiency indicates the percentage of reactive collisions. The best-fitting software gmkin (GK)^57^ was also employed to analyze the kinetic data, thus obtaining *k*′ values superimposable with the ones from the semilog plots.

### Computational Details

Guess structures of [T3-H]^−^ and [rT3-H]^−^ were obtained, combining chemical intuition with a preliminary conformational search using the tool Conformer Distribution, as implemented in the software suite Spartan’16,^58^ and the semiempirical method PM6. Subsequently, geometries were optimized at the B3LYP level using the 6-311+G** basis set for light atoms and def2TZVP for iodine. Harmonic vibrational frequencies were computed at the same level to obtain IR spectra and thermodynamic corrections to the electronic energies. [T3-H]^−^ and [rT3-H]^−^ isomers were reoptimized with the B3LYP-D3 functional using the same basis set. Thermodynamic corrections were also calculated using the dispersion-corrected functional B3LYP-D3. In addition, single-point energy calculations at the MP2 level were performed on the B3LYP-D3 optimized structures to compare the relative energies obtained by DFT with the ones computed with an *ab initio* method. To obtain relative enthalpies and Gibbs free energies at the MP2 level, B3LYP-D3 thermodynamic corrections were used. The whole set of quantum mechanics calculations were performed using the Gaussian 09 Rev. D01 package.^59^ Calculated IR spectra reported in this work were all obtained at the B3LYP/6-311+G** (I = def2TZVP) level and were scaled by a 0.978 factor to compensate the well-known bias of hybrid DFT functionals to overestimate harmonic vibrational frequencies. This value is consistent with the one used in previous works on similar compounds.^35,60^ CCSs were calculated from the B3LYP-D3 optimized structures with the MobCal software as appropriately modified by Kim et al. for mobility separations employing N_2_ as drift gas.^48,61^

## Results and Discussion

### Tandem Mass Spectrometry and Ion Spectroscopy

Deprotonated triiodothyronine, [T3-H]^−^, and reverse triiodothyronine, [rT3-H]^−^, (see Scheme 1) were mass selected and assayed using CID and IRMPD. The resulting (photo)dissociation mass spectra are reported in Figures S1 and S2 in the Supporting Information (SI). Photofragmentation products are consistent with CID ones, and both experiments replicate the fragmentation pattern already reported in the literature.^28,29^ [T3-H]^−^ is characterized by an important loss of NH_3_ (*m*/*z* 632.7), which is also observed in deprotonated tyrosine, thyronine, and T2.^29^ In addition, characteristic fragments are observed at *m*/*z* 448.9, attributable to the loss of HI accompanied by HNCHCOOH, and at *m*/*z* 126.9, which is consistent with the iodide anion. These fragmentation products agree with a preferential deprotonation on the carboxylic acid function of T3, which is followed by proton transfer from the aromatic CH in ortho*-*position to iodine and loss of the corresponding iodide.^29^ In the case of [rT3-H]^−^, the fragmentation spectrum shows a remarkably different scenario considering the structural similarity with its isomer [T3-H]^−^. In particular, the fragment at *m*/z 632.7, *i.e*., loss of ammonia, is a minor feature in the CID spectrum of [rT3-H]^−^ (Figure S1). Also, the product ion at *m*/*z* 448.9 is lower in abundance while additional fragments appear. Particularly interesting are the ions at *m*/*z* 575.8 and 359.8. The dissociation mechanisms proposed by Blanksby et al. for their formation implies that [rT3-H]^−^ mainly exists in the phenoxide form.^29^ Thus, different deprotonation sites can be supposed for [T3-H]^−^ and [rT3-H]^−^. Indeed, an appropriate tool for obtaining the structural features of the two ions is necessary to confirm this hypothesis.

**Figure 1:**
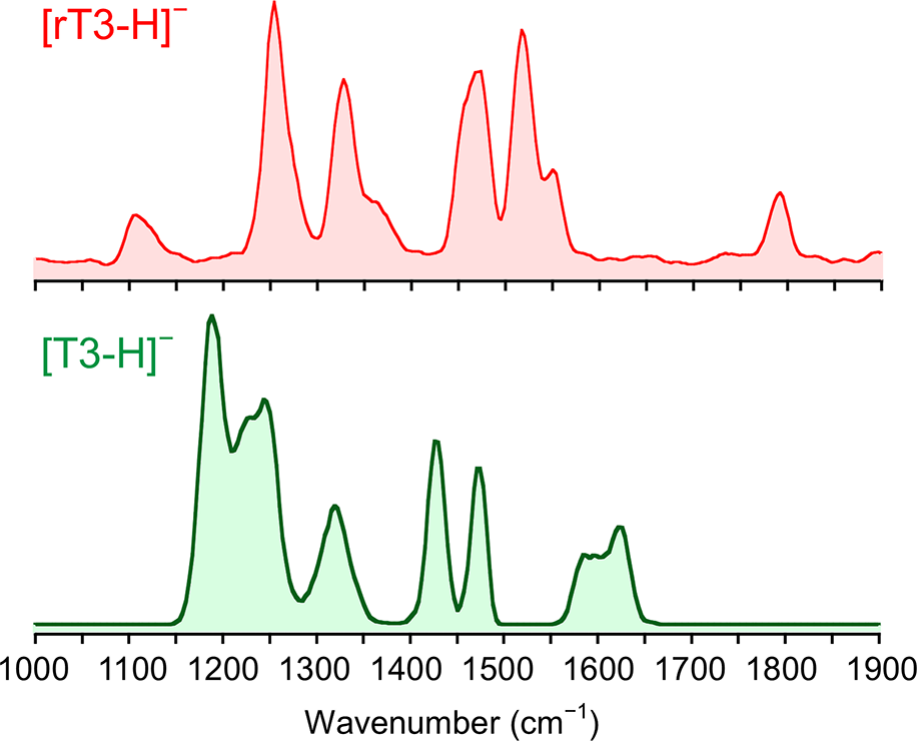
IRMPD spectra of [T3-H]^−^ and [rT3-H]^−^ in the fingerprint range.

IRMPD spectra of [T3-H]^−^ and [rT3-H]^−^ are reported in Figure 1, while the corresponding photofragmentation mass spectra are shown in Figure S2 in the SI.

IRMPD spectra of [T3-H]^−^ and [rT3-H]^−^ are noticeably different considering that the two species are structural isomers that differ only for the position of one among three iodine atoms. What immediately stands out is the activity in the range above 1500 cm^–1^. In particular, [T3-H]^−^ presents a broad band around 1600 cm^–1^, while no significant absorptions rise in the same region for [rT3-H]^−^ that is instead active at ca. 1780 cm^–1^. The band at 1780 cm^–1^ can be arguably attributed to the CO stretching of a free carboxylic acid,^35,62,63^ suggesting predominant deprotonation of rT3 on the phenolic group. On the contrary, the [T3-H]^−^ absorption at 1600 cm^–1^ is characteristic of asymmetric carboxylate stretching,^33,36^ thus pointing to deprotonation of the carboxylic moiety. Eventually, more information on the two isomeric structures is still hiding under the signals in the lower wavenumber range of the reported spectra. A computational survey of the isomers of [T3-H]^−^ and [rT3-H]^−^ deprotonated either at the carboxylic or phenolic group was therefore performed to correlate the spectroscopic data to specific vibrational modes.

### Conformational Sampling of [T3-H]^−^ and [rT3-H]^−^

**Figure 2:**
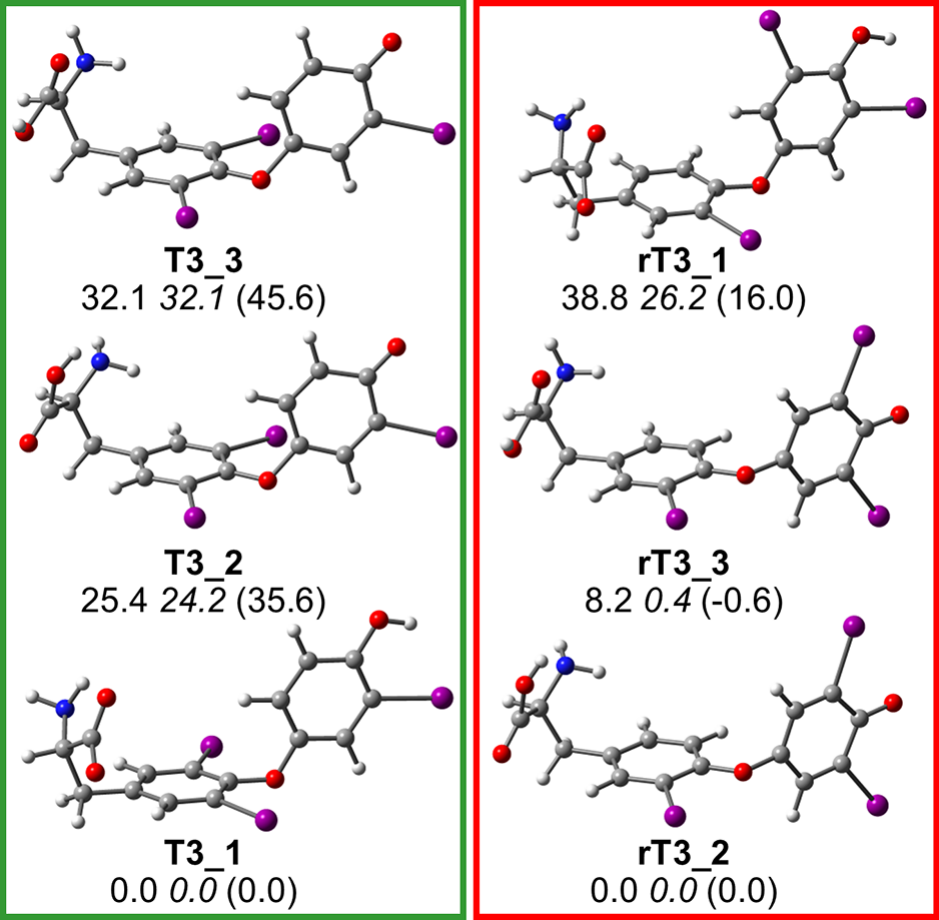
Optimized geometries of [T3-H]^−^ (left panel) and [rT3-H]^−^ (right panel) isomers. Relative free energies at 298 K at the B3LYP, B3LYP-D3, and (MP2) levels are provided in kJ mol^–1^.

Figure 2 reports optimized geometries and Gibbs free energies of [T3-H]^−^ and [rT3-H]^−^. Additional thermodynamic data can be found in Table S1 in the SI. Two isomer families are represented for each ion, in particular, **T3_1** and **rT3_1** show deprotonation on the carboxylic moiety, while conformers **T3_2** and **T3_3** and **rT3_2** and **rT3_3** are in the phenolate form. Relative Gibbs free energies are reported at the B3LYP, B3LYP-D3, and MP2 levels. All the calculation methods agree on indicating [T3-H]^−^ to be more stable in carboxylate form while [rT3-H]^−^ in the phenolate one. The dispersion-corrected functional and MP2 are consistent in suggesting the comparable energy of the two conformations of phenolate rT3, i.e., **rT3_2** and **rT3_3**, while B3LYP overestimates the Gibbs free energy of **rT3_3**. The same does not apply, however, to **T3_2** and **T3_3**. Indeed, the whole set of computed energies shows that **T3_3** is higher in energy than **T3_2** by ca. 9 kJ mol^–1^. Henceforth, B3LYP-D3 free energies will be used as the reference unless otherwise specified.

The lowest-lying isomer of [T3-H]^−^ is **T3_1**, in which the carboxylate functionality interacts with one of the amino hydrogen atoms (*r*_O9...H–N_ = 2.04 Å) and the phenolic H is oriented toward the iodine atom of the B ring (*r*_OH...I_ = 2.63 Å). **T3_2** lays 24.2 kJ mol^–1^ higher in free energy than **T3_1**, likely due to the lower acidity of the phenol functionality, and presents a H-bond between the carboxylic H, in *anti* configuration, and the amino nitrogen. This structural motif is characteristic of amino acids not bearing the charge on the α-aminocarboxylic portion.^35,64,65^**T3_3** is found at 32.1 kJ mol^–1^ and shares the phenolate motif with **T3_2** while differing for the aminoacidic moiety conformation. In fact, a *syn* carboxylic acid shows a H-bond between the carbonyl oxygen and an amino hydrogen (*r*_O...HN_ = 2.60 Å). The computational survey of [rT3-H]^−^ reveals a rather different picture. It is possible to recognize the same structural motifs of the [T3-H]^−^ conformers. In fact, **rT3_1** presents the negative charge on the carboxylate, while **rT3_2** and **rT3_3** are in the phenolate form, with the carboxylic acid in either *anti* or *syn* configuration, respectively. However, thermodynamic data point to the prevalence of the phenolate isomers **rT3_2** and **rT3_3**, which are almost isoenergetic and thus likely to coexist in the gas-phase population. In contrast, the carboxylate structure **rT3_1** shows a higher free energy, estimated at 26.2 kJ mol^–1^. To summarize, [T3-H]^−^ is likely to present a negatively charged carboxylate group, while [rT3-H]^−^ should mainly exist in a phenolate form.

### Vibrational Mode Assignment

**Figure 3:**
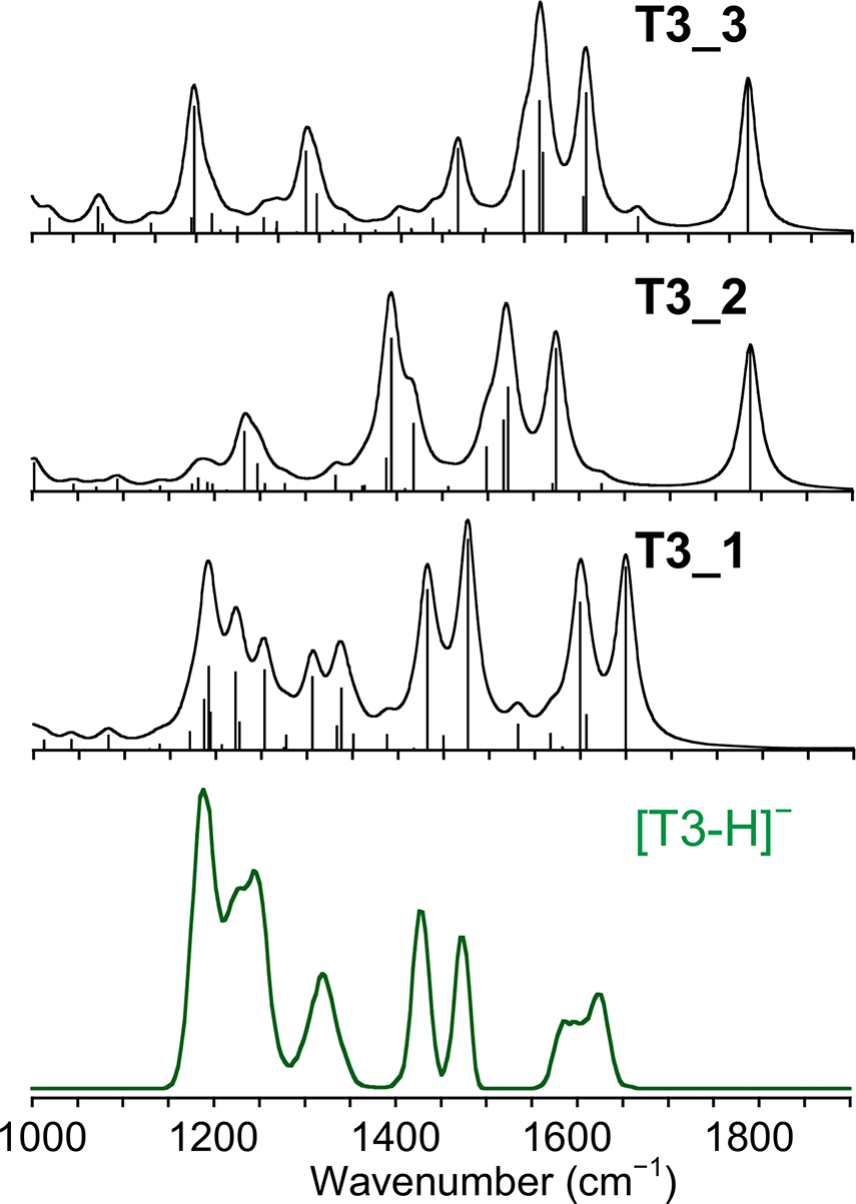
Experimental IRMPD spectrum (green profile, bottom) of [T3-H]^−^ compared with harmonic IR spectra of **T3_1**, **T3_2**, and **T3_3** calculated at the B3LYP/6–311+G** (I = def2TZVP) level and scaled by a factor of 0.978. The scale of the *y*-axis is the same for all theoretical IR spectra.

To confirm the landscape emerging from thermodynamic results, experimental IRMPD spectra of [T3-H]^−^ and [rT3-H]^−^ were compared to the theoretical ones calculated for the candidate isomers. A comparison of the IRMPD spectrum of [T3-H]^−^ with the calculated IR spectra of **T3_1**, **T3_2**, and **T3_3** is reported in Figure 3, while the assigned vibrational modes are described in Table S2.

**Figure 4:**
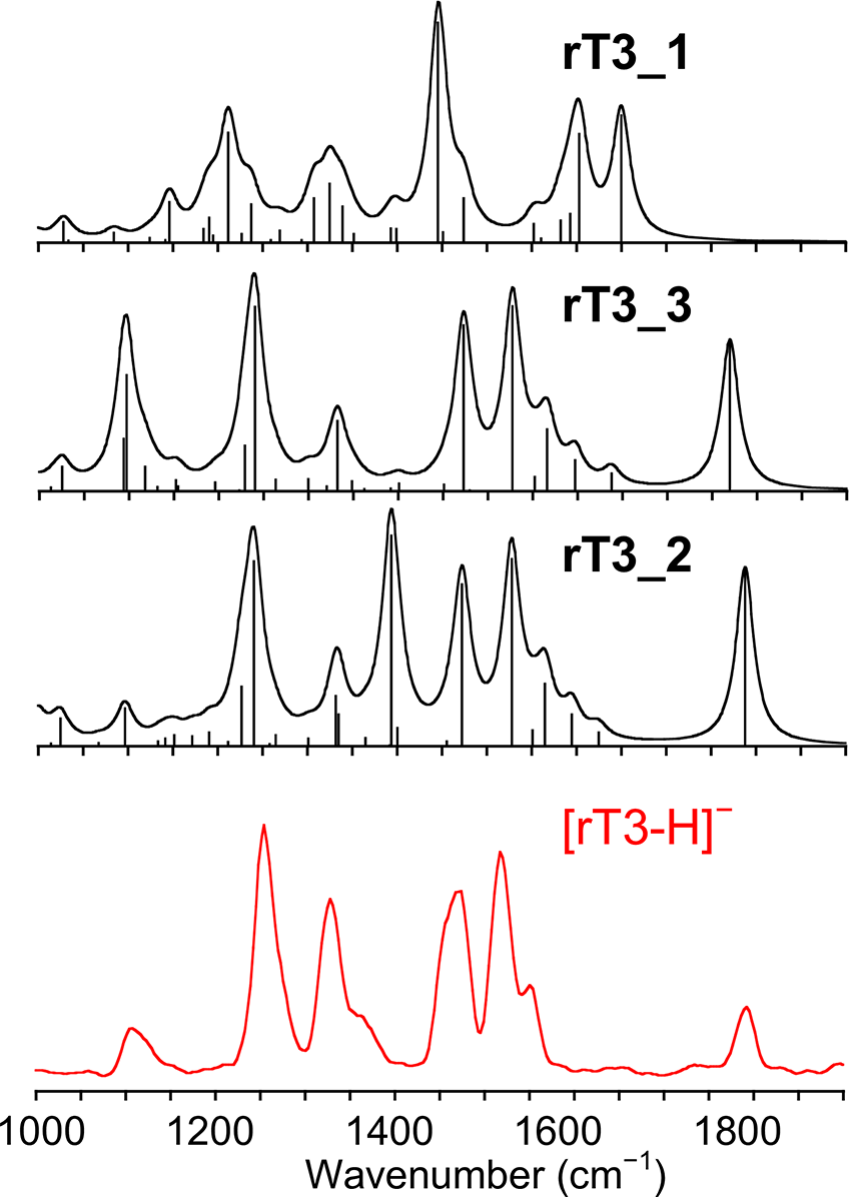
Experimental IRMPD spectrum (red profile, bottom) of [rT3-H]^−^ compared with harmonic IR spectra of **rT3_2**, **rT3_3**, and **rT3_1** calculated at the B3LYP/6-311+G** (I = def2TZVP) level and scaled by a factor of 0.978. The scale of the *y*-axis is the same for all theoretical IR spectra.

The IRMPD spectrum is fairly matched by the one pertaining to **T3_1**, in agreement with the trend of calculated free energies. The spectral range above 1600 cm^–1^ is highly informative. In particular, the couple of merged bands at 1589 and 1625 cm^–1^ is well interpreted by the carboxylate asymmetric stretching and NH_2_ scissoring calculated for **T3_1** at 1650 and 1600 cm^–1^, respectively. In addition, the IRMPD spectrum lacks any activity in the 1750–1800 cm^–1^ range, where the asymmetric stretching of the carboxylic acid functionality in **T3_2** and **T3_3** is expected. The couple of experimental bands at 1428 and 1478 cm^–1^ can be attributed to **T3_1** vibrational modes calculated at 1432 (C4–O stretching coupled with ring A C–H bending) and 1477 cm^–1^ (C4′–O stretching coupled with ring B C–H bending), respectively. The low wavenumber range of the spectrum is characterized by the presence of broad and poorly resolved absorptions between 1190 and 1325 cm^–1^, which are well interpreted by the calculated vibrations of **T3_1** in that region. Moving on, Figure 4 shows the IRMPD spectrum of [rT3-H]^−^ to be compared with the theoretical IR spectra of **rT3_2**, **rT3_3**, and **rT3_1**.

Vibrational modes are reported in Table S3 in the SI. Above 1600 cm^–1^, the [rT3-H]^−^ experimental spectrum is markedly different when compared to the one of [T3-H]^−^. A single band rises at 1793 cm^–1^, well matching the carboxylic acid asymmetric stretch calculated for both **rT3_2** and **rT3_3** at ca. 1790 cm^–1^. This vibration is characteristic of the neutral carboxylic acid portion and confirms that deprotonation of rT3 occurs at the level of the phenol group. Moreover, there is no activity in the 1600–1700 cm^–1^ range where **rT3_1**, on the contrary, presents bands pertaining to the carboxylate moiety. Interestingly, the position of the C4′–O stretch is also indicative of the predominant presence of **rT3_2** and **rT3_3** in the sampled gas-phase population. Indeed, the phenolate isomers present a characteristic vibrational mode at ca. 1528 cm^–1^, in correspondence with a strong experimental band at 1527 cm^–1^. Conversely, the same mode is calculated for **rT3_1** at 1446 cm^–1^, which does not well match any IRMPD feature. Finally, it is not possible to clearly discriminate between the **rT3_2** and **rT3_3** conformers just on the basis of spectroscopic data. In fact, the only signal characteristic of either one of the two structures is the carboxylic OH bend, which is calculated at 1394 and 1095 cm^–1^ for **rT3_2** and **rT3_3**, respectively. Given that, the low activity of [rT3-H]^−^ in the 1400 cm^–1^ region could, at a first sight, suggest the predominance of **rT3_3**, although several examples in the literature point to a poor IRMPD activity of the carboxylic OH bend when strongly interacting with a H-bond acceptor group, such as the amino nitrogen.^33,64−66^ Thus, the small shoulder at 1370 cm^–1^ can be likely attributed to **rT3_2**. Finally, the experimental band at 1107 cm^–1^, which agrees with both the OH bend calculated at 1095 cm^–1^ for **rT3_3** and ring B breathing mode expected at 1097 cm^–1^ for **rT3_2**, does not concur to an unambiguous assignment among the two candidates.

### Ion Mobility Analyses

**Figure 5:**
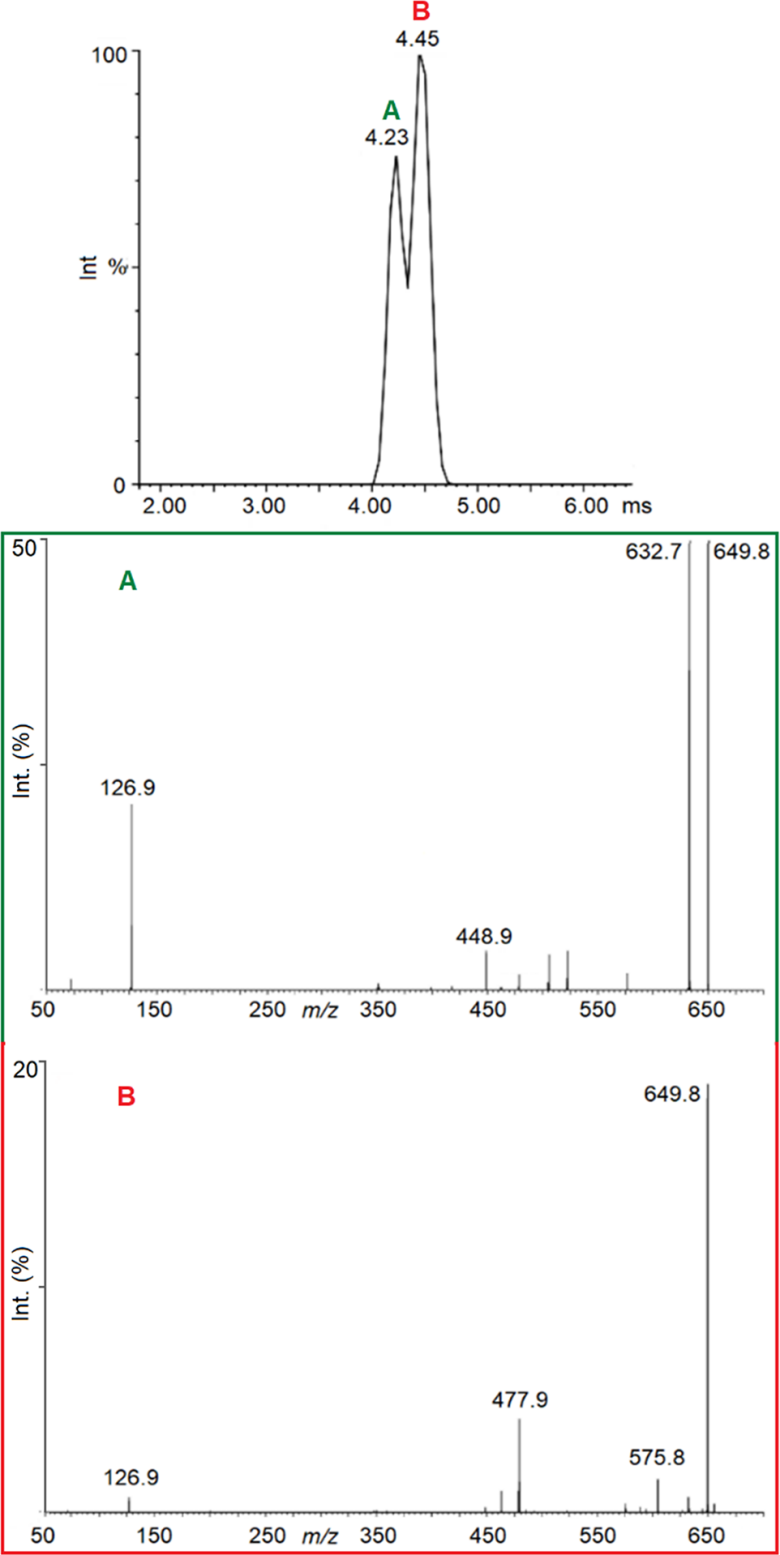
Top: mobilogram extracted for *m*/*z* 649.8 recording the isomer mixture. Bottom: MS/MS spectra in correspondence to the mobilogram peaks at (A) 4.23 ms and (B) 4.45 ms.

Ion mobility experiments, ESI(−)–IMS, were carried out on standard solutions of both compounds. Drift time values recorded for [T3-H]^−^ and [rT3-H]^−^ were 4.18 and 4.34 ms, respectively (Figure S3). These results indicate a different surface exposure for the two anions and suggest the possibility to separate them based on their gas-phase mobility. Therefore, an equimolar mixture of T3 and rT3 in methanol was analyzed using ESI(−)–IMS under the same experimental conditions employed for the single isomer samples. The ion-extracted mobilogram of *m*/*z* 649.8 showed two drift times values at 4.23 and 4.45 ms, respectively (see Figure 5).

Based on the drift times obtained for the single compounds, one can assign the peak at 4.23 ms to [T3-H]^−^ and the one at 4.45 ms to [rT3-H]^−^. A slight deviation is observed between the drift times recorded in the mixed solution and those of the pure compounds (Figure S3) likely ascribable to the overlapping of [T3-H]^−^ and [rT3-H]^−^ peaks in the mobilogram of the mixture, causing some displacement of their respective maxima. To confirm these assumptions, ESI–IMS-MS/MS experiments were performed on the ion at *m*/*z* 649.8, which was mass isolated from the mobilogram of the mixture solution. The ions were fragmented using the collision cell placed after the mobility unit, thus obtaining CID spectra for each recorded drift time. As shown in Figure 5, the CID spectrum of the *m*/*z* 649.8 ion extracted from the peak with the lower drift time presents the typical fragmentation pattern of [T3-H]^−^, whereas the one from the higher drift time peak shares the fragmentation spectrum of [rT3-H]^−^. Relative abundances of the fragments may be different when compared to the CID spectra of Figure S1 due to the higher fragmentation yields achieved in the trap, even when operating with the same CE. Finally, the obtained drift times were correlated with the CCS of the two isomers.^48^ The so-obtained CCS values are reported in Table 1 and are 209 and 215 Å for [T3-H]^−^ and [rT3-H]^−^, respectively. Therefore, [T3-H]^−^ is confirmed to have a slightly tighter structure than [rT3-H]^−^. Experimental values are compared with theoretical ones obtained from the B3LYP-D3-optimized structures to support the structural attributions based on the spectroscopic data and propose a rationale for the observed ion-mobility distinct behavior of [T3-H]^−^ and [rT3-H]^−^. Indeed, Table 1 reports a good agreement between the experimental CCS of [T3-H]^−^ and the computed value for **T3_1**, while the higher CCS value of [rT3-H]^−^ is in good agreement with the simulated ones of the phenoxide isomers **rT3_2** and **rT3_3**, in particular with the last one. Therefore, [T3-H]^−^ and [rT3-H]^−^ are confirmed to exist mainly in carboxylate and phenolate forms, respectively. In addition, for both isomers, the carboxylate structures (**T3_1** and **rT3_1**) present lower calculated CCS values than the phenolate ones (**T3_2**,**3** and **rT3_2**,**3**), suggesting the localization of the negative charge in the anions to be the crucial factor for the different mobility behavior of [T3-H]^−^ and [rT3-H]^−^.

**Table 1:** Calculated CCS Values for **T3_1–3**, and **rT3_1–3** Compared with the Experimental CCS Values Obtained from the Drift Times of [T3-H]^−^ and [rT3-H]^−^

	CCS (Å^2^)	
	theo	exp
**T3_1**	208	209
**T3_2**	240	
**T3_3**	237	
**rT3_2**	224	215
**rT3_3**	219	
**rT3_1**	209	

### Ion-Molecule Reactions

[T3-H]^−^ and [rT3-H]^−^ were mass analyzed in the cell of an FT-ICR, which allowed measurement of the accurate mass of the anions, obtaining an *m*/z value of 649.78319 with a mass error of 0.66 ppm with respect to the exact mass of the formula [C_15_H_11_NO_4_I_3_]^−^ (649.78276 Da). [T3-H]^−^ and [rT3-H]^−^ were allowed to react with selected neutral molecules with appropriate volatility aiming to substantiate the different deprotonation sites of the two anions, possibly enabling characteristic ion-molecule reactions.^67,68^ In this regard, Kenttämaa et al. showed diethylmethoxyborane (DEMB) to react selectively with the phenolate functionality, producing the DEMB adduct, while carboxylate groups showed no reactivity.^42^ Based on this evidence, both [T3-H]^−^ and [rT3-H]^−^ were mass isolated and stored in the presence of DEMB introduced at a stationary pressure of 10^–7^ mbar. Even though the reaction time spanned from 10 to 180 s, both anions showed no reactivity with DEMB. However, this result does not invalidate the attribution of [rT3-H]^−^ to the phenoxide isomer. In fact, the presence of two bulky electron-withdrawing (EW) substituents in both ortho-positions to the phenolate function could hinder the formation of the adduct. Such an effect is noted by Kenttämaa et al. for deprotonated vanillin, where the presence of an EW aldehyde group in para*-*position to the phenol inhibits the formation of the adduct.^42^

Additional experiments were designed to obtain information on the acid properties of T3 and rT3. First, theoretical gas-phase acidity (Δ*G*_acid_) values were obtained from calculations at the B3LYP-D3 level by summing the Gibbs free energy of the proton, as reported by York et al.,^69^ to the one of the most stable deprotonated hormones, either **T3_1** or **rT3_2**, and finally subtracting the Gibbs free energies of neutral T3 and rT3, respectively. Optimized structures and thermodynamic data of neutral thyroid hormones are reported in Figure S4 and Table S1 in the SI, respectively. Calculations show rT3 to be a stronger acid (Δ*G*_acid_(rT3) = 1326 kJ mol^–1^) than T3 (Δ*G*_acid_(T3) = 1345 kJ mol^–1^). The relatively low Δ*G*_acid_ values of both species indicate strong acidic properties. Both [T3-H]^−^ and [rT3-H]^−^ were allowed to react with acetylacetone and TFA, which present Δ*G*_acid_ values of 1409 and 1328 kJ mol^–1^, respectively.^70,71^ Acetylacetone showed no reactivity with either [T3-H]^−^ or [rT3-H]^−^ in agreement with its higher Δ*G*_acid_. On the other hand, two competitive products were formed by the reaction of TFA with the two deprotonated thyroid hormones, namely [TFA-H]^−^ by proton abstraction and [(r)T3 + TFA-H]^−^ by addition. Mass spectra of the two anions reacting with TFA recorded at a 2 s reaction time are reported in Figure S5 in the SI, kinetic plots are shown in Figure S6, and tabulated kinetic data are reported in Table 2. The proton transfer from TFA to [T3-H]^−^ shows an efficiency of 22%, slightly higher than the one for the same reaction with [rT3-H]^−^ (10%). Eventually, the higher reaction efficiency obtained for [T3-H]^−^ is consistent with the less pronounced acidity of T3 when compared with the rT3 isomer.

**Table 2:** Kinetic Data for the Proton Transfer Reaction [X-H]^−^ + TFA → X + [TFA-H]^−^

X	*k*_exp_^a^	Eff (%)^b^^,^^c^
T3	2.53	22
rT3	1.12	10

a Second-order rate constant in units of 10^–10^ cm^3^ s^–1^ at 298 K, estimated error: ± 30%.

b Eff = *k*_exp_/*k*_ADO_ × 100, where *k*_ADO_ is 1.14 × 10^–9^.^55,56^

c Proton transfer reaction is accompanied by TFA addition, yielding [(r)T3 + TFA-H]^−^ (Eff (%) = 7 and 4 for X = T3 and rT3, respectively).

## Conclusions

The isomeric T3 and rT3 thyroid hormones were analyzed as bare deprotonated species, employing a selection of mass spectrometry-based techniques to obtain structural and reactivity information of potential interest for isomer discrimination. Calculations at the DFT and MP2 levels were performed to rationalize the experimental data. An unambiguous characterization of the favored deprotonation sites of [T3-H]^−^ and [rT3-H]^−^ was obtained, combining IRMPD spectroscopy and DFT calculations of candidate structures and associated vibrational frequencies. [T3-H]^−^ was found to exist predominantly in the carboxylate form, while [rT3-H]^−^ showed spectral features characteristic of a phenolate anion. This distinct behavior agrees with the increased acidity of the phenol group due to the substitution of the hydrogen atom in ortho*-*position with an iodine atom. Spectroscopic data well correlate with fragmentation behavior, which shows different product ions for the two thyroid hormones. Ion mobilities of [T3-H]^−^ and [rT3-H]^−^ were assayed, obtaining promising results for the establishment of novel separation procedures based on drift times for thyroid hormones. Indeed, the two anions showed different arrival times in a TWIMS sector, which are associated with experimental CCS values in N_2_ of 209 and 215 Å for [T3-H]^−^ and [rT3-H]^−^, respectively. Calculated CCSs were obtained and found to be in good agreement with the experimental data. Furthermore, the theoretical CCSs of carboxylate isomers were found to be consistently lower than the ones of the phenolate structures, suggesting a strong influence of the different negative charge localization in [T3-H]^−^ and [rT3-H]^−^ on their ion mobilities. Finally, ion-molecule reactions indicate a stronger acidity of rT3 compared with the one of T3 in agreement with the calculated Δ*G*_acid_ of T3 and rT3 obtained at the B3LYP-D3 level, *i.e.*, 1345 and 1326 kJ mol^–1^, respectively.

To conclude, the present work represents the first experimental assessment of different deprotonation sites within T3 and rT3, involving either the carboxylic or phenol groups, respectively. This feature may not only affect the gas-phase behavior of the two anions, driving their unimolecular reactivity and mobility, but can also supposedly have an impact in the mirror physiological activity of the two isomeric thyroid hormones.

## Supporting Information Available

Figures of (photo)fragmentation mass spectra, mobilograms, IMR data, tables of IRMPD absorptions, and calculated vibrational frequencies (PDF)

## References

[ref1] Rousset B.; Dupuy C.; Miot F.; Dumont J. Chapter 2 Thyroid Hormone Synthesis and Secretion; Endotext: 2000. 25905405

[ref2] Sinha R. A.; Singh B. K.; Yen P. M. Thyroid Hormone Regulation of Hepatic Lipid and Carbohydrate Metabolism. Trends Endocrinol. Metab. 2014, 25, 538–545. 10.1016/j.tem.2014.07.001. 25127738

[ref3] Yen P. M. Physiological and Molecular Basis of Thyroid Hormone Action. Physiol. Rev. 2001, 81, 1097–1142. 10.1152/physrev.2001.81.3.1097. 11427693

[ref4] Zhang J.; Lazar M. A. The Mechanism of Action of Thyroid Hormones. Annu. Rev. Physiol. 2000, 62, 439–466. 10.1146/annurev.physiol.62.1.439. 10845098

[ref5] Köhrle J.; Spanka M.; Irmscher K.; Hesch R. D. Flavonoid Effects on Transport, Metabolism and Action of Thyroid Hormones. Prog. Clin. Biol. Res. 1988, 280, 323–340. 3140249

[ref6] Kelly G. S. Peripheral Metabolism of Thyroid Hormones: A Review. Altern. Med. Rev. 2000, 5, 306–333. 10956378

[ref7] Devereaux D.; Tewelde S. Z. Hyperthyroidism and Thyrotoxicosis. Emerg. Med. Clin. North Am. 2014, 32, 277–292. 10.1016/j.emc.2013.12.001. 24766932

[ref8] Ross D. S.; Burch H. B.; Cooper D. S.; Greenlee M. C.; Laurberg P.; Maia A. L.; Rivkees S. A.; Samuels M.; Sosa J. A.; Stan M. N.; Walter M. A. 2016 American Thyroid Association Guidelines for Diagnosis and Management of Hyperthyroidism and Other Causes of Thyrotoxicosis. Thyroid 2016, 26, 1343–1421. 10.1089/thy.2016.0229. 27521067

[ref9] Klein I.; Ojamaa K. Thyroid Hormone and the Cardiovascular System. N. Engl. J. Med. 2001, 344, 501–509. 10.1056/NEJM200102153440707. 11172193

[ref10] Gilbert J. Hypothyroidism. Medicine 2017, 45, 506–509. 10.1016/j.mpmed.2017.05.009.

[ref11] Chaker L.; Bianco A. C.; Jonklaas J.; Peeters R. P. Hypothyroidism. Lancet 2017, 390, 1550–1562. 10.1016/S0140-6736(17)30703-1. 28336049 PMC6619426

[ref12] Biondi B.; Cooper D. S. The Clinical Significance of Subclinical Thyroid Dysfunction. Endocr. Rev. 2008, 29, 76–131. 10.1210/er.2006-0043. 17991805

[ref13] Jones D. D.; May K. E.; Geraci S. A. Subclinical Thyroid Disease. Am. J. Med. 2010, 123, 502–504. 10.1016/j.amjmed.2009.12.023. 20569751

[ref14] Sánchez-Carbayo M.; Mauri M.; Alfayate R.; Miralles C.; Soria F. Analytical and Clinical Evaluation of TSH and Thyroid Hormones by Electrochemiluminescent Immunoassays. Clin. Biochem. 1999, 32, 395–403. 10.1016/S0009-9120(99)00032-6. 10667473

[ref15] Soldin O. P.; Hilakivi-Clarke L.; Weiderpass E.; Soldin S. J. Trimester-Specific Reference Intervals for Thyroxine and Triiodothyronine in Pregnancy in Iodine-Sufficient Women Using Isotope Dilution Tandem Mass Spectrometry and Immunoassays. Clin. Chim. Acta 2004, 349, 181–189. 10.1016/j.cccn.2004.06.021. 15469872 PMC3625638

[ref16] Kazerouni F.; Amirrasouli H. Performance Characteristics of Three Automated Immunoassays for Thyroid Hormones. Casp. J. Intern. Med. 2012, 3, 400–404. PMC3861902 24358433

[ref17] Welsh K. J.; Soldin S. J. DIAGNOSIS OF ENDOCRINE DISEASE: How reliable are free thyroid and total T3 hormone assays?. Eur. J. Endocrinol. 2016, 175, R255–R263. 10.1530/EJE-16-0193. 27737898 PMC5113291

[ref18] Bowerbank S. L.; Carlin M. G.; Dean J. R. A Direct Comparison of Liquid Chromatography-Mass Spectrometry with Clinical Routine Testing Immunoassay Methods for the Detection and Quantification of Thyroid Hormones in Blood Serum. Anal. Bioanal. Chem. 2019, 411, 2839–2853. 10.1007/s00216-019-01724-2. 31079177 PMC6522465

[ref19] Scientific T. TriiodothyronineT3 competitive ELISA method; [Available from: https://www.thermofisher.com/order/catalog/ product/EIAT3C.

[ref20] Scientific T. Thyroxine T4 competitive ELISA method; [Available from: https://www.thermofisher.com/order/catalog/product/ EIAT4C?SID=srch-srp-EIAT4C.

[ref21] Wu A. H. B.; French D. Implementation of Liquid Chromatography/Mass Spectrometry into the Clinical Laboratory. Clin. Chim. Acta 2013, 420, 4–10. 10.1016/j.cca.2012.10.026. 23085380

[ref22] Yong S.; Chen Y.; Lee T. K.; Lee H. K. Determination of Total Thyroxine in Human Serum by Hollow Fiber Liquid-Phase Microextraction and Liquid Chromatography-Tandem Mass Spectrometry. Talanta 2014, 126, 163–169. 10.1016/j.talanta.2014.03.058. 24881548

[ref23] Jonklaas J.; Sathasivam A.; Wang H.; Gu J.; Burman K. D.; Soldin S. J. Total and Free Thyroxine and Triiodothyronine: Measurement Discrepancies, Particularly in Inpatients. Clin. Biochem. 2014, 47, 1272–1278. 10.1016/j.clinbiochem.2014.06.007. 24936679 PMC4187214

[ref24] Tai S. S. C.; Bedner M.; Phinney K. W. Development of a Candidate Reference Measurement Procedure for the Determination of 25-Hydroxyvitamin D3 and 25-Hydroxyvitamin D 2 in Human Serum Using Isotope-Dilution Liquid Chromatography Tandem Mass Spectrometry. Anal. Chem. 2010, 82, 1942–1948. 10.1021/ac9026862. 20136128 PMC2838390

[ref25] Tai S. S. C.; Sniegoski L. T.; Welch M. J. Candidate Reference Method for Total Thyroxine in Human Serum: Use of Isotope-Dilution Liquid Chromatography - Mass Spectrometry with Electrospray Ionization. Clin. Chem. 2002, 48, 637–642. 10.1093/clinchem/48.4.637. 11901062

[ref26] Soukhova N.; Soldin O. P.; Soldin S. J. Isotope Dilution Tandem Mass Spectrometric Method for T4/T3. Clin. Chim. Acta 2004, 343, 185–190. 10.1016/j.cccn.2004.01.007. 15115693 PMC3634919

[ref27] Kumar A. P.; Jin H.; Jo S. C.; Kim C.; Nam S. H.; Lee Y. I. Isomeric Discrimination and Quantification of Thyroid Hormones, T3 and RT3, by the Single Ratio Kinetic Method Using Electrospray Ionization Mass Spectrometry. J. Am. Soc. Mass Spectrom. 2010, 21, 14–22. 10.1016/j.jasms.2009.07.004. 19853471

[ref28] Zhang Y.; Conrad A. H.; Conrad G. W. Detection and Quantification of 3,5,3′-Triiodothyronine and 3,3′,5′-Triiodothyronine by Electrospray Ionization Tandem Mass Spectrometry. J. Am. Soc. Mass Spectrom. 2005, 16, 1781–1786. 10.1016/j.jasms.2005.06.017. 16182556

[ref29] Couldwell A. M.; Thomas M. C.; Mitchell T. W.; Hulbert A. J.; Blanksby S. J. Tandem Mass Spectrometry of Deprotonated Iodothyronines. Rapid Commun. Mass Spectrom. 2005, 19, 2295–2304. 10.1002/rcm.2061. 16021613

[ref30] Eyler J. R. Infrared Multiple Photon Dissociation Spectroscopy of Ions in Penning Traps. Mass Spectrom. Rev. 2009, 28, 448–467. 10.1002/mas.20217. 19219931

[ref31] Polfer N. C. Infrared Multiple Photon Dissociation Spectroscopy of Trapped Ions. Chem. Soc. Rev. 2011, 40, 2211–2221. 10.1039/c0cs00171f. 21286594

[ref32] Martens J.; van Outersterp R. E.; Vreeken R. J.; Cuyckens F.; Coene K. L. M.; Engelke U. F.; Kluijtmans L. A. J.; Wevers R. A.; Buydens L. M. C.; Redlich B.; Berden G.; Oomens J. Infrared Ion Spectroscopy: New Opportunities for Small-Molecule Identification in Mass Spectrometry - A Tutorial Perspective. Anal. Chim. Acta 2020, 1093, 1–15. 10.1016/j.aca.2019.10.043. 31735202

[ref33] Oomens J.; Steill J. D.; Redlich B. Gas-Phase IR Spectroscopy of Deprotonated Amino Acids. J. Am. Chem. Soc. 2009, 131, 4310–4319. 10.1021/ja807615v. 19267428

[ref34] Scuderi D.; Correia C. F.; Balaj O. P.; Ohanessian G.; Lemaire J.; Maitre P. Structural Characterization by IRMPD Spectroscopy and DFT Calculations of Deprotonated Phosphorylated Amino Acids in the Gas Phase. ChemPhysChem 2009, 10, 1630–1641. 10.1002/cphc.200800856. 19347918

[ref35] Corinti D.; Gregori B.; Guidoni L.; Scuderi D.; McMahon T. B.; Chiavarino B.; Fornarini S.; Crestoni M. E. Complexation of Halide Ions to Tyrosine: Role of Non-Covalent Interactions Evidenced by IRMPD Spectroscopy. Phys. Chem. Chem. Phys. 2018, 20, 4429–4441. 10.1039/C7CP06657K. 29372198

[ref36] Corinti D.; Chiavarino B.; Scuderi D.; Fraschetti C.; Filippi A.; Fornarini S.; Crestoni M. E. Molecular Properties of Bare and Microhydrated Vitamin B5–Calcium Complexes. Int. J. Mol. Sci. 2021, 22, 692 10.3390/ijms22020692. PMC7826572 33445631

[ref37] Corinti D.; Coletti C.; Re N.; Paciotti R.; Maître P.; Chiavarino B.; Crestoni M. E.; Fornarini S. Short-Lived Intermediates (Encounter Complexes) in Cisplatin Ligand Exchange Elucidated by Infrared Ion Spectroscopy. Int. J. Mass Spectrom. 2019, 435, 7–17. 10.1016/j.ijms.2018.10.012.

[ref38] Corinti D.; Paciotti R.; Re N.; Coletti C.; Chiavarino B.; Crestoni M. E.; Fornarini S. Binding Motifs of Cisplatin Interaction with Simple Biomolecules and Aminoacid Targets Probed by IR Ion Spectroscopy. Pure Appl. Chem. 2020, 92, 3–13. 10.1515/pac-2019-0110.

[ref39] Schröder D.; Buděšínský M.; Roithová J. Deprotonation of P-Hydroxybenzoic Acid: Does Electrospray Ionization Sample Solution or Gas-Phase Structures?. J. Am. Chem. Soc. 2012, 134, 15897–15905. 10.1021/ja3060589. 22931167

[ref40] Patrick A. L.; Cismesia A. P.; Tesler L. F.; Polfer N. C. Effects of ESI Conditions on Kinetic Trapping of the Solution-Phase Protonation Isomer of p-Aminobenzoic Acid in the Gas Phase. Int. J. Mass Spectrom. 2017, 418, 148–155. 10.1016/j.ijms.2016.09.022. 28781574 PMC5542407

[ref41] Sinha R. K.; Scuderi D.; Maitre P.; Chiavarino B.; Crestoni M. E.; Fornarini S. Elusive Sulfurous Acid: Gas-Phase Basicity and IR Signature of the Protonated Species. J. Phys. Chem. Lett. 2015, 6, 1605–1610. 10.1021/acs.jpclett.5b00450. 26263321

[ref42] Zhu H.; Max J. P.; Marcum C. L.; Luo H.; Abu-Omar M. M.; Kenttämaa H. I. Identification of the Phenol Functionality in Deprotonated Monomeric and Dimeric Lignin Degradation Products via Tandem Mass Spectrometry Based on Ion–Molecule Reactions with Diethylmethoxyborane. J. Am. Soc. Mass Spectrom. 2016, 27, 1813–1823. 10.1007/s13361-016-1442-9. 27553243

[ref43] Beegle L. W.; Kanik I.; Matz L.; Hill H. H. Electrospray Ionization High-Resolution Ion Mobility Spectrometry for the Detection of Organic Compounds, 1. Amino Acids. Anal. Chem. 2001, 73, 3028–3034. 10.1021/ac001519g. 11467550

[ref44] Johnson P. V.; Kim H. I.; Beegle L. W.; Kanik I. Electrospray Ionization Ion Mobility Spectrometry of Amino Acids: Ion Mobilities and a Mass-Mobility Correlation. J. Phys. Chem. A 2004, 108, 5785–5792. 10.1021/jp0492117. 16834170

[ref45] Gidden J.; Bowers M. T. Gas-Phase Conformational and Energetic Properties of Deprotonated Dinucleotides. Eur. Phys. J. D 2002, 20, 409–419. 10.1140/epjd/e2002-00170-7.

[ref46] Corinti D.; Maccelli A.; Crestoni M. E.; Cesa S.; Quaglio D.; Botta B.; Ingallina C.; Mannina L.; Tintaru A.; Chiavarino B.; Fornarini S. IR Ion Spectroscopy in a Combined Approach with MS/MS and IM-MS to Discriminate Epimeric Anthocyanin Glycosides (Cyanidin 3-O-Glucoside and -Galactoside). Int. J. Mass Spectrom. 2019, 444, 116179 10.1016/j.ijms.2019.116179.

[ref47] Sinclair E.; Hollywood K. A.; Yan C.; Blankley R.; Breitling R.; Barran P. Mobilising Ion Mobility Mass Spectrometry for Metabolomics. Analyst 2018, 143, 4783–4788. 10.1039/c8an00902c. 30209461

[ref48] Campuzano I.; Bush M. F.; Robinson C. V.; Beaumont C.; Richardson K.; Kim H.; Kim H. I. Structural Characterization of Drug-like Compounds by Ion Mobility Mass Spectrometry: Comparison of Theoretical and Experimentally Derived Nitrogen Collision Cross Sections. Anal. Chem. 2012, 84, 1026–1033. 10.1021/ac202625t. 22141445

[ref49] Lemaire J.; Boissel P.; Heninger M.; Mauclaire G.; Bellec G.; Mestdagh H.; Simon A.; Le Caer S.; Ortega J. M.; Glotin F.; Maitre P.; Le Caer S.; Ortega J. M.; Glotin F.; Maitre P. Gas Phase Infrared Spectroscopy of Selectively Prepared Ions. Phys. Rev. Lett. 2002, 89, 273001–273002. 10.1103/PhysRevLett.89.273002. 12513202

[ref50] Bakker J. M.; Besson T.; Lemaire J.; Scuderi D.; Maître P. Gas-Phase Structure of a π-Allyl–Palladium Complex: Efficient Infrared Spectroscopy in a 7 T Fourier Transform Mass Spectrometer. J. Phys. Chem. A 2007, 111, 13415–13424. 10.1021/jp074935e. 18052260

[ref51] Prell J. S.; O’Brien J. T.; Williams E. R. IRPD Spectroscopy and Ensemble Measurements: Effects of Different Data Acquisition and Analysis Methods. J. Am. Soc. Mass Spectrom. 2010, 21, 800–809. 10.1016/j.jasms.2010.01.010. 20185332

[ref52] Angelelli F.; Chiavarino B.; Crestoni M. E.; Fornarini S. Binding of Gaseous Fe(III)-Heme Cation to Model Biological Molecules: Direct Association and Ligand Transfer Reactions. J. Am. Soc. Mass Spectrom. 2005, 16, 589–598. 10.1016/j.jasms.2005.01.011. 15792729

[ref53] Corinti D.; Coletti C.; Re N.; Piccirillo S.; Giampà M.; Crestoni M. E.; Fornarini S. Hydrolysis of Cis- and Transplatin: Structure and Reactivity of the Aqua Complexes in a Solvent Free Environment. RSC Adv. 2017, 7, 15877–15884. 10.1039/C7RA01182B.

[ref54] Lanucara F.; Crestoni M. E. Biomimetic Oxidation Reactions of a Naked Manganese(V)-Oxo Porphyrin Complex. Chem. - A Eur. J. 2011, 17, 12092–12100. 10.1002/chem.201101432. 21905135

[ref55] Su T.; Su E. C. F.; Bowers M. T. Ion-Polar Molecule Collisions. Conservation of Angular Momentum in the Average Dipole Orientation Theory. The AADO Theory. J. Chem. Phys. 1978, 69, 2243–2250. 10.1063/1.436783.

[ref56] Su T.; Chesnavich W. J. Parametrization of the Ion–Polar Molecule Collision Rate Constant by Trajectory Calculations. J. Chem. Phys. 1982, 76, 5183–5185. 10.1063/1.442828.

[ref57] https://cran.r-project.org/web/packages/mkin/index.html Version 0.9.50.2 Accessed 01 Sept. 2020.

[ref58] Spartan 16, Program for Calculation of Molecular Properties; Wavefuntion Inc: Irvine, CA, USA, 2016**.**

[ref59] Frisch M. J.; Trucks G. W.; Schlegel H. B.; Scuseria G. E.; Robb M. A.; Cheeseman J. R.; Scalmani G.; Barone V.; Mennucci B.; Petersson G. A.; Nakatsuji H.; Caricato M.; Li X.; Hratchian H. P.; Izmaylov A. F.; Bloino J.; Zheng G.; Sonnenberg J. L.; Hada M.; Ehara M.; Toyota K.; Fukuda R.; Hasegawa J.; Ishida M.; Nakajima T.; Honda Y.; Kitao O.; Nakai H.; Vreven T.; Montgomery J. A. Jr.; Peralta J. E.; Ogliaro F.; Bearpark M.; Heyd J. J.; Brothers E.; Kudin K. N.; Staroverov V. N.; Keith T.; Kobayashi R.; Normand J.; Raghavachari K.; Rendell A.; Burant J. C.; Iyengar S. S.; Tomasi J.; Cossi M.; Rega N.; Millam J. M.; Klene M.; Knox J. E.; Cross J. B.; Bakken V.; Adamo C.; Jaramillo J.; Gomperts R.; Stratmann R. E.; Yazyev O.; Austin A. J.; Cammi R.; Pomelli C.; Ochterski J. W.; Martin R. L.; Morokuma K.; Zakrzewski V. G.; Voth G. A.; Salvador P.; Dannenberg J. J.; Dapprich S.; Daniels A. D.; Farkas Ö.; Foresman J. B.; Ortiz J. V.; Cioslowski J.; Fox D. J.: Gaussian 09, revision D.01, Gaussian, Inc.: Wallingford, CT, 2010

[ref60] Burt M.; Wilson K.; Marta R.; Hasan M.; Scott Hopkins W.; McMahon T. Assessing the Impact of Anion−π Effects on Phenylalanine Ion Structures Using IRMPD Spectroscopy. Phys. Chem. Chem. Phys. 2014, 16, 24223–24234. 10.1039/C4CP03776F. 25294414

[ref61] Kim H.; Kim H. I.; Johnson P. V.; Beegle L. W.; Beauchamp J. L.; Goddard W. A.; Kanik I. Experimental and Theoretical Investigation into the Correlation between Mass and Ion Mobility for Choline and Other Ammonium Cations in N2. Anal. Chem. 2008, 80, 1928–1936. 10.1021/ac701888e. 18278882

[ref62] Wu R.; McMahon T. B. An Investigation of Protonation Sites and Conformations of Protonated Amino Acids by IRMPD Spectroscopy. ChemPhysChem 2008, 9, 2826–2835. 10.1002/cphc.200800543. 18846594

[ref63] Wang Y.; Alhajji E.; Rieul B.; Berthias F.; Maître P. Infrared Isomer-Specific Fragmentation for the Identification of Aminobutyric Acid Isomers Separated by Differential Mobility Spectrometry. Int. J. Mass Spectrom. 2019, 443, 16–21. 10.1016/j.ijms.2019.05.014.

[ref64] Corinti D.; De Petris A.; Coletti C.; Re N.; Chiavarino B.; Crestoni M. E.; Fornarini S. Cisplatin Primary Complex with L-Histidine Target Revealed by IR Multiple Photon Dissociation (IRMPD) Spectroscopy. ChemPhysChem 2017, 18, 318–325. 10.1002/cphc.201601172. 27935248

[ref65] Paciotti R.; Corinti D.; De Petris A.; Ciavardini A.; Piccirillo S.; Coletti C.; Re N.; Maitre P.; Bellina B.; Barran P.; Chiavarino B.; Crestoni M. E.; Fornarini S. Cisplatin and Transplatin Interaction with Methionine: Bonding Motifs Assayed by Vibrational Spectroscopy in the Isolated Ionic Complexes. Phys. Chem. Chem. Phys. 2017, 19, 26697–26707. 10.1039/C7CP05203K. 28876340

[ref66] Chiavarino B.; Sinha R. K.; Crestoni M. E.; Corinti D.; Filippi A.; Fraschetti C.; Scuderi D.; Maitre P.; Fornarini S. Binding Motifs in the Naked Complexes of Target Amino Acids with an Excerpt of Antitumor Active Biomolecule: An Ion Vibrational Spectroscopy Assay. Chem. – A Eur. J. 2021, 27, 2348–2360. 10.1002/chem.202003555. 33175428

[ref67] Osburn S.; Ryzhov V. Ion–Molecule Reactions: Analytical and Structural Tool. Anal. Chem. 2013, 85, 769–778. 10.1021/ac302920a. 23077968

[ref68] Liu J. K.; Niyonsaba E.; Alzarieni K. Z.; Boulos V. M.; Yerabolu R.; Kenttämaa H. I. Determination of the Compound Class and Functional Groups in Protonated Analytes via Diagnostic Gas-phase Ion-molecule Reactions. Mass Spectrom. Rev. 2021, mas.21727 10.1002/mas.21727. 34435381

[ref69] Moser A.; Range K.; York D. M. Accurate Proton Affinity and Gas-Phase Basicity Values for Molecules Important in Biocatalysis. J. Phys. Chem. B 2010, 114, 13911–13921. 10.1021/jp107450n. 20942500 PMC2970571

[ref70] Caldwell G.; Renneboog R.; Kebarle P. Gas Phase Acidities of Aliphatic Carboxylic Acids, Based on Measurements of Proton Transfer Equilibria. Can. J. Chem. 1989, 67, 611–618. 10.1139/v89-092.

[ref71] Taft R. W.; Bordwell F. G. Structural and Solvent Effects Evaluated from Acidities Measured in Dimethyl Sulfoxide and in the Gas Phase. Acc. Chem. Res. 1988, 21, 463–469. 10.1021/ar00156a005.

