# Supplementary material for: Molecular Basis for the Remarkably Different Gas-Phase Behavior of Deprotonated Thyroid Hormones Triiodothyronine (T3) and Reverse Triiodothyronine (rT3): A Clue for Their Discrimination?
Source: Anal Chem. 2021 Oct 29;93(44):14869–77. doi: 10.1021/acs.analchem.1c03892 (PMC8581966; doi:10.1021/acs.analchem.1c03892)
Supplement: Supplementary file 1 — ac1c03892_si_001.pdf [file ac1c03892_si_001.pdf]

Supporting Information for

Molecular basis for the remarkably different gas-phase behavior of deprotonated thyroid hormones triiodothyronine (T3) and reverse triiodothyronine (rT3): a clue for their discrimination?

Davide Corinti[a]*, Barbara Chiavarino[a], Mattia Spano[a], Aura Tintaru[b], Simonetta Fornarini[a], Maria Elisa Crestoni[a]*

[a] Dipartimento di Chimica e Tecnologie del Farmaco, Università di Roma "La Sapienza", I-00185, Roma, Italy; [b] Aix Marseille Univ, CNRS, Institut de Chimie Radicalaire, UMR 7273, 13397, Marseille, France.

* davide.corinti@uniroma1.it
* mariaelisa.crestoni@uniroma1.it

Figure S1. ESI (-)-MS/MS spectra obtained from solutions of T3 (top) and rT3 (bottom) with a trap collision energy of 30 eV (laboratory frame)

Figure S2. A) Photofragmentation mass spectrum of $[T3\text{-}H]^-$ at 1240 cm^{-1}. B) Photofragmentation mass spectrum of $[rT3\text{-}H]^-$ at 1467 cm^{-1}.

Figure S3. Ion-extracted mobilograms of m/z 649.7 of T3 (top) and rT3 (bottom), respectively.

Figure S4. Optimized structures of A) **T3-neu** and B) **rT3-neu** at the B3LYP-D3 level.

Figure S5. Mass spectra of $[T3\text{-}H]^-$ and $[rT3\text{-}H]^-$ after 2 second reaction time with TFA at 6 10^{-8} mbar. Both spectra show $[(r)T3\text{-}H]^-$ at *m/z* 650, $[(r)T3\text{+}TFA\text{-}H]^-$ at *m/z* 764, $[TFA\text{-}H]^-$ at *m/z* 113 and $[(TFA)_2\text{-}H]^-$ at *m/z* 227.

Figure S6. Time dependence of relative ion abundances for the reaction of A) $[T3\text{-}H]^-$ (*m/z* 650) and B) $[rT3\text{-}H]^-$ (*m/z* 650) with TFA forming $[(r)T3\text{+}TFA\text{-}H]^-$ (*m/z* 764) and $[TFA\text{-}H]^-$ (*m/z* 113) in the FT-ICR cell at the TFA pressure of 10^{-7} mbar. The abundance of $[(TFA)_2\text{-}H]^-$ (*m/z* 227) was summed to the one of $[TFA\text{-}H]^-$.

Table S1. Thermodynamic data for the optimized structures of $[T3\text{-}H]^-$, $[rT3\text{-}H]^-$, T3 and rT3.

Table S2. Observed IRMPD resonances of $[T3\text{-}H]^-$ and calculated vibrational frequencies for the lowest lying isomers **T3_1** and **T3_2**.

Table S3. Observed IRMPD resonances of $[rT3\text{-}H]^-$ and calculated vibrational frequencies for the lowest lying isomers **rT3_1** and **rT3_2**.

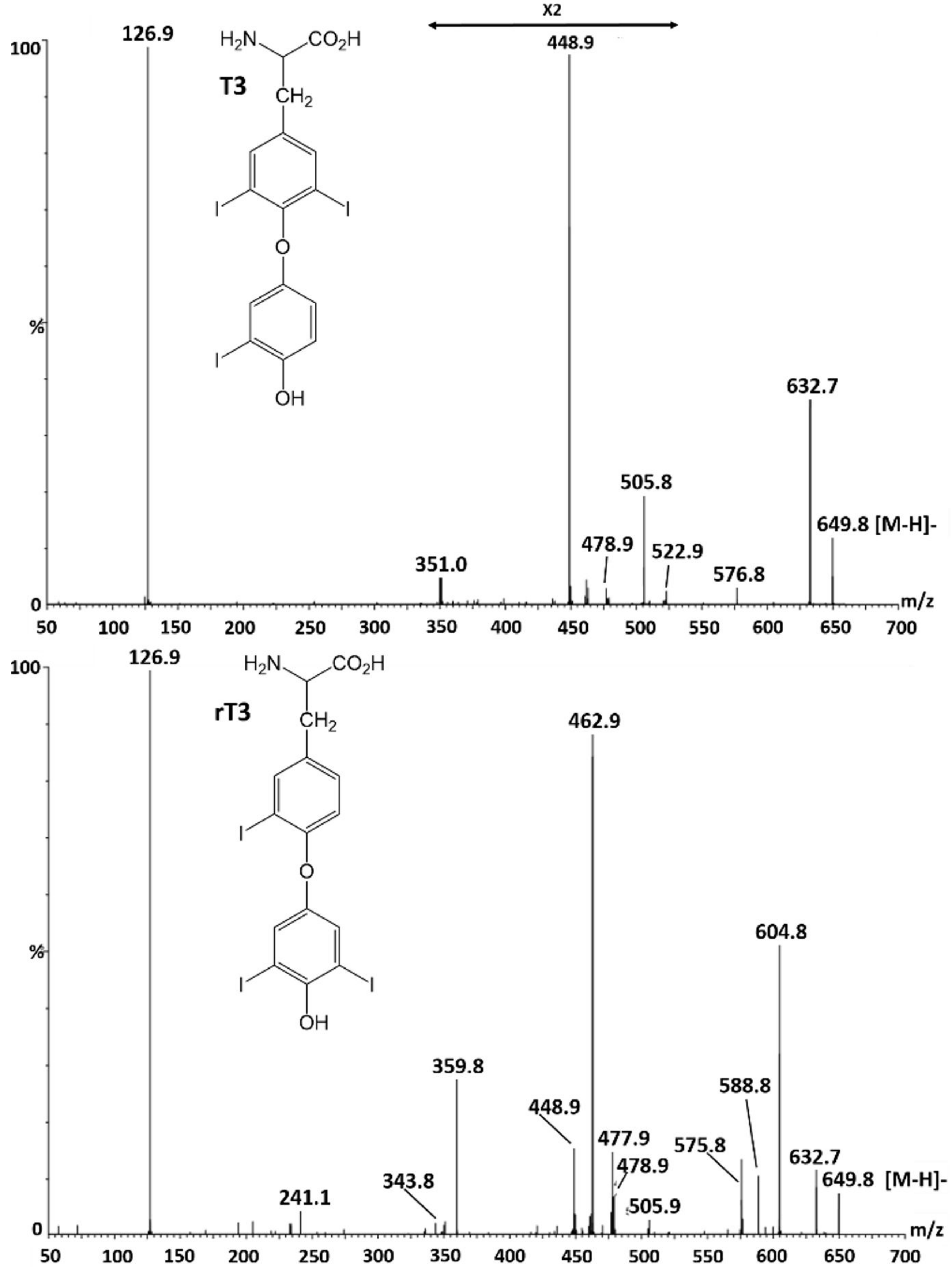

Figure S1. ESI (-)-MS/MS spectra obtained from solutions of T3 (top) and rT3(bottom) with a trap collision energy of 30 eV (laboratory frame)

A)

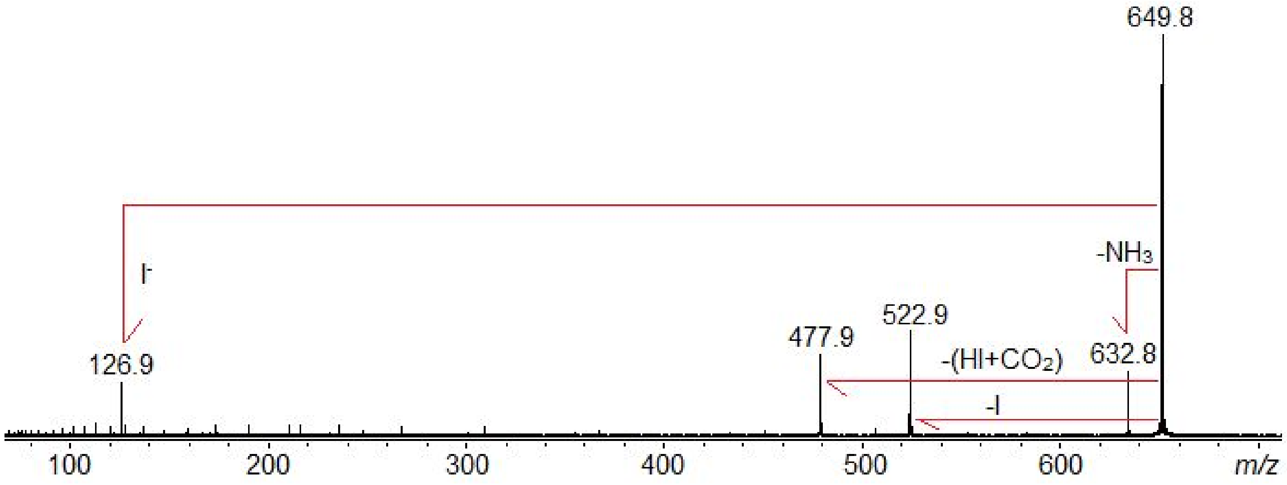

B)

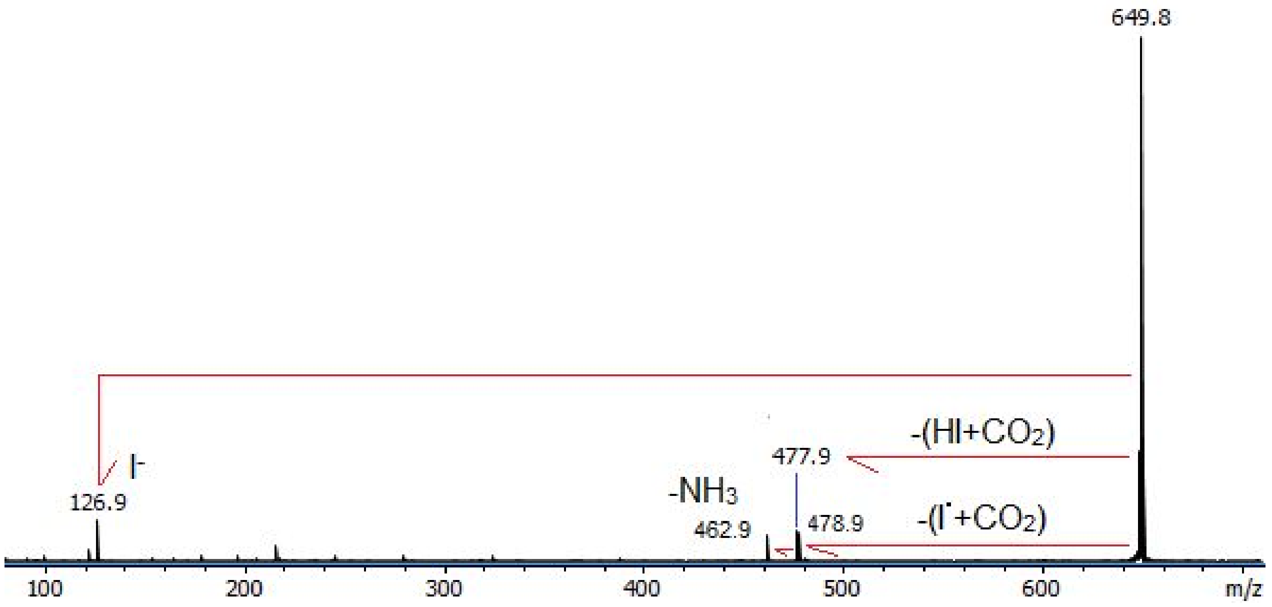

Figure S2. A) Photofragmentation mass spectrum of $[T3\text{-}H]^-$ at 1240 cm^{-1}. B) Photofragmentation mass spectrum of $[rT3\text{-}H]^-$ at 1467 cm^{-1}.

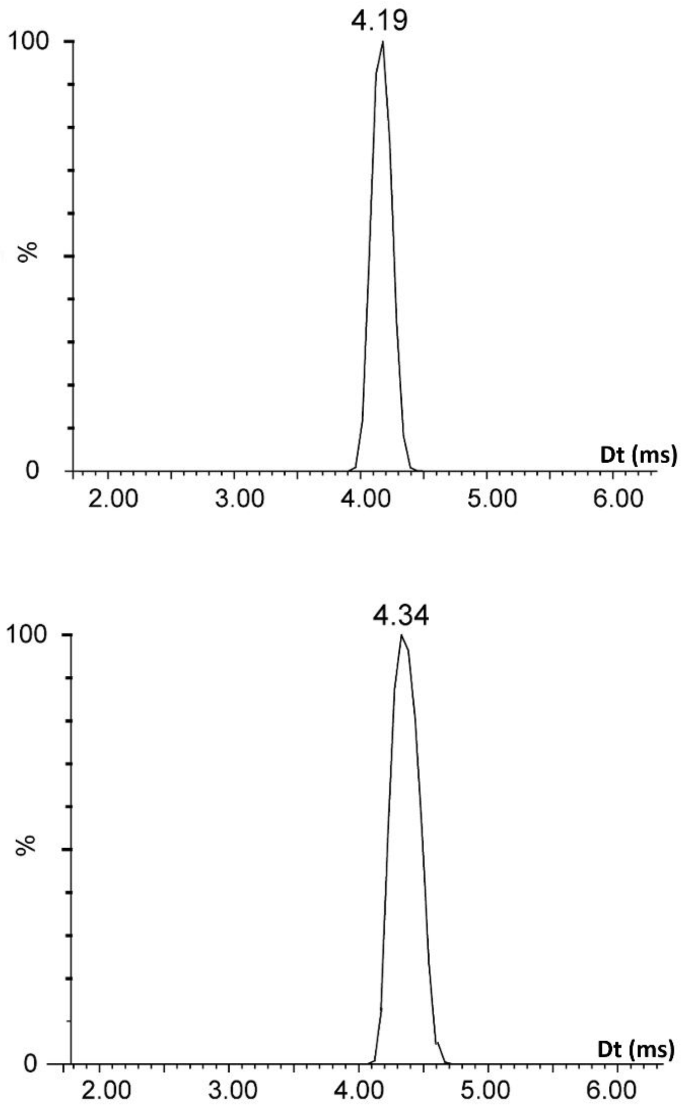

Figure S3. Ion-extracted mobilograms of m/z 649.7 of T3 (top) and rT3 (bottom), respectively.

A)

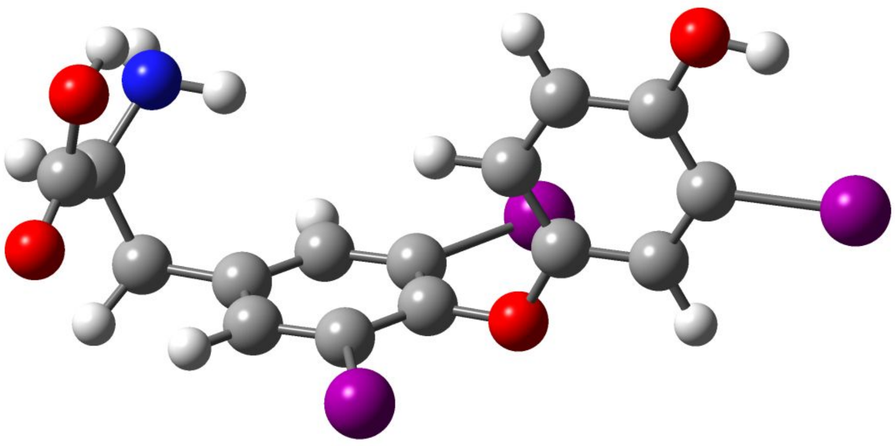

B)

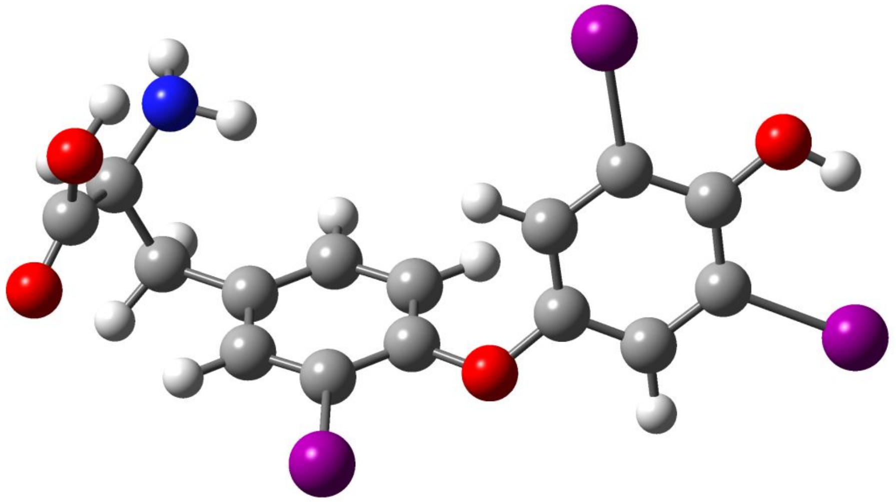

Figure S4. Optimized structures of A) **T3-neu** and B) **rT3-neu** at the B3LYP-D3 level.

A)

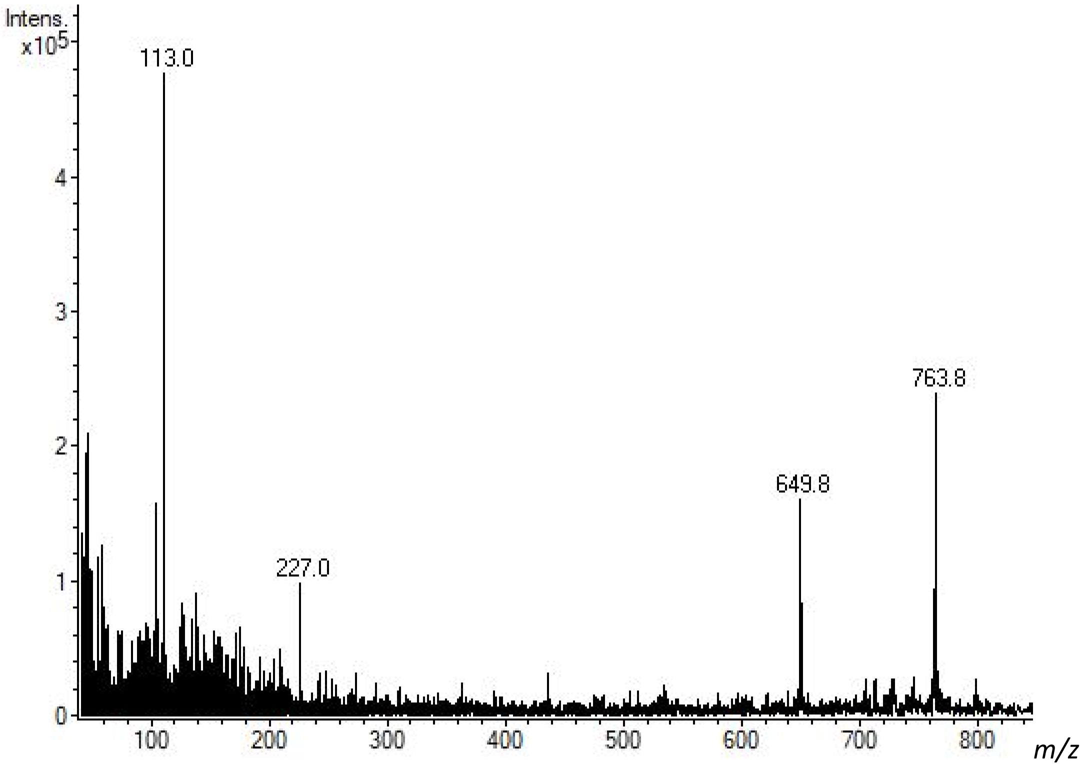

B)

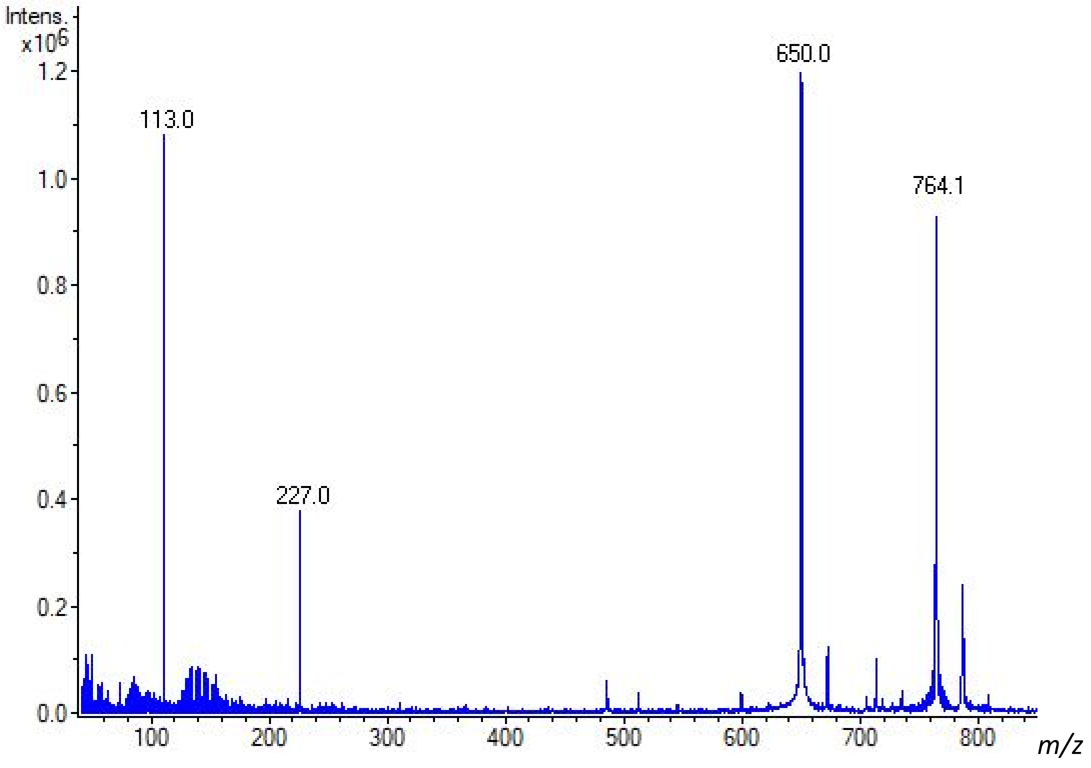

Figure S5. Mass spectra of A) [T3-H]$^-$ and B) [rT3-H]$^-$ after 2 second reaction time with TFA. Both spectra show [(r)T3-H]$^-$ at *m/z* 650, [(r)T3+TFA-H]$^-$ at *m/z* 764, [TFA-H]$^-$ at *m/z* 113 and [(TFA)$_2$-H]$^-$ at *m/z* 227.

A)

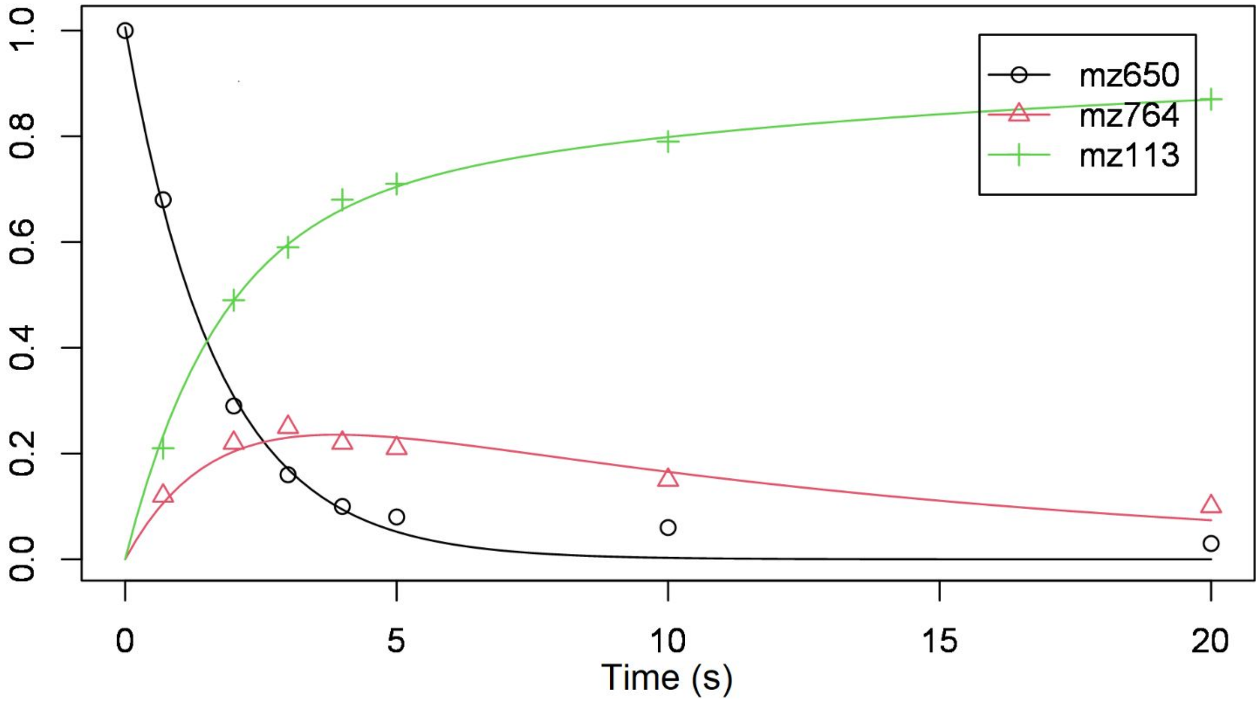

B)

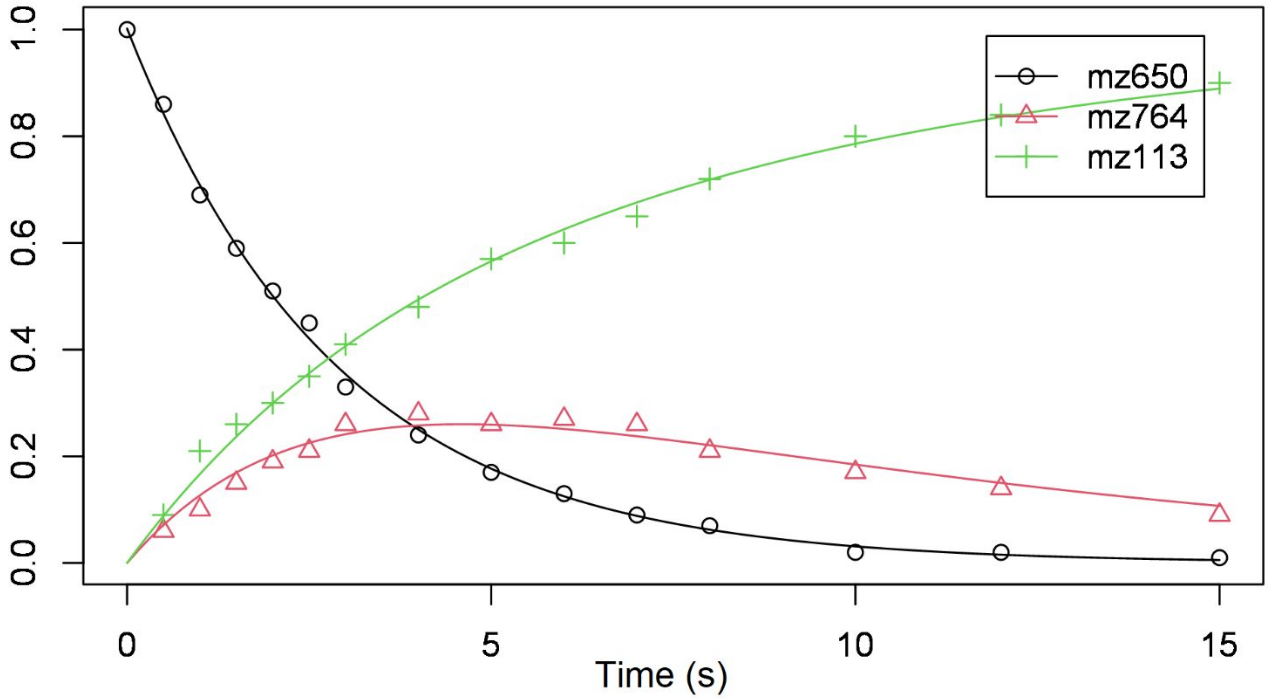

Figure S6. Time dependence of relative ion abundances for the reaction of A) $[T3\text{-}H]^-$ (*m/z* 650) and B) $[rT3\text{-}H]^-$ (*m/z* 650) with TFA forming $[(r)T3\text{+}TFA\text{-}H]^-$ (*m/z* 764) and $[TFA\text{-}H]^-$ (*m/z* 113) in the FT-ICR cell at the TFA pressure of 10^{-7} mbar. The abundance of $[(TFA)_2\text{-}H]^-$ (*m/z* 227) was summed to the one of $[TFA\text{-}H]^-$.

Table S1. Thermodynamic data for the optimized structures of $[T3\text{-}H]^-$, $[rT3\text{-}H]^-$, T3 and rT3.

	B3LYP				B3LYP-D3				MP2			
	H^a	G^b	H_{rel}^c	G_{rel}^c	H^a	G^b	H_{rel}^c	G_{rel}^c	$H^{a,d}$	$G^{b,d}$	$H_{rel}^{c,d}$	$G_{rel}^{c,d}$
**T3_1**	-1827.339771	-1827.423739	0.0	0.0	-1827.388701	-1827.470187	0.0	0.0	-1823.53023	-1823.611716	0.0	0.0
**T3_2**	-1827.330515	-1827.414047	24.3	25.4	-1827.377833	-1827.460966	28.5	24.2	-1823.771068	-1823.51504	39.9	35.6
**T3_3**	-1827.326849	-1827.411527	33.9	32.1	-1827.374284	-1827.457942	37.9	32.1	-1823.767762	-1823.511843	48.3	42.6
**rT3_1**	-1827.336341	-1827.421079	39.3	38.8	-1827.381253	-1827.465133	36.4	26.2	-1823.519059	-1823.602939	26.2	16.0
**rT3_2**	-1827.35132	-1827.435854	0.0	0.0	-1827.395123	-1827.475103	0.0	0.0	-1823.784816	-1823.52904	0.0	0.0
**rT3_3**	-1827.347638	-1827.432744	9.7	8.2	-1827.390747	-1827.474929	11.5	0.5	-1823.781678	-1823.525083	10.4	-0.6
**T3-neu**	-1827.860793	-1827.944465	-	-	-1827.908703	-1827.992350	-	-	-1824.046800	-1824.130447	-	-
**rT3-neu**	-1827.862551	-1827.946765	-	-	-1827.906385	-1827.990150	-	-	-1824.041962	-1824.125727	-	-

[a]Enthalpy corrected electronic energies in Hartree particle^{-1}; [b] Free energy corrected electronic energies in Hartree particle^{-1}; [c] in kJ mol^{-1}; Thermodynamic corrections from B3LYP-D3 harmonic frequency calculations.

Table S2. Observed IRMPD resonances of $[T3\text{-}H]^-$ and calculated vibrational frequencies for the lowest lying calculated isomers **T3_1** and **T3_2**.

		**[T3-H]**$^-$	
**Experimental**[a]	**Calculated**[a,b]		**Vibrational mode**
	**T3_1**	**T3_2**	
		1023 (57)	NH2 wag + CH_2 sciss
1191	1188 (71)		phenol O-H stretch + ring A C-H bends + C1'-C7 stretch
	1193 (118)		phenol O-H stretch + CH_2 twist
1248	1222 (110)		C4-O stretch + both ring C-H bends
		1233 (120)	C2'-H bend
		1248 (54)	ring B C-H bends
	1254 (114)		C1'-O stretch + ring B C-H bends
1325	1307 (103)		CH_2 wag + NH_2 twist
	1338 (88)		CH_2 wag + C-H bend
		1389 (66)	CH_2 twist + ring A C-H bend
		1394 (311)	carboxylic O-H bend
		1418 (136)	C4-O stretch + ring A C-H bends
1428	1432 (228)		C4-O stretch + ring A C-H bends
1478	1477 (300)		C4'-O stretch + ring B C-H bends
		1499 (90)	C1'-C2' stretch + C1'-C6' stretch
		1518 (144)	C4'-O stretch + ring A breath + ring B breath
		1523 (211)	C4'-O stretch + ring A breath + ring B breath
		1575 (290)	C4'-O stretch + C2'-C3' stretch + C5'-C6' stretch + ring B C-H bends
1589	1600 (209)		NH_2 sciss
	1607 (50)		ring B breath
1625	1650 (260)		CO_2 asym stretch + NH_2 sciss
		1788 (296)	CO_2 asym stretch + carboxylic O-H bend

[a] in cm^{-1}. [b] intensities in brackets in km mol^{-1}. Vibrations with intensities lower than 50 km mol^{-1} are not reported.

Table S3. Observed IRMPD resonances of [rT3-H]$^-$ and calculated vibrational frequencies for the lowest lying calculated isomers **rT3_1** and **rT3_2**.

[rT3-H]$^-$			
**Experimental**[a]	**Calculated**[a,b]		**Vibrational mode**
	**rT3_2**	**rT3_1**	
1107	1097 (62)		C3'-C4' stretch + C4'-C5' stretch + C2'-H bend + C6'-H bend
		1146 (84)	phenol O-H stretch + C2'-H bend + C5'-H bend
		1190 (52)	C2-H bend + C6-H bend + C1'-C7 stretch
		1212 (227)	C4-O stretch + C2'-H bend + ring A breath
		1238 (79)	phenol O-H bend + ring B breath
1257	1227 (99)		C2'H bend + C6'H bend
	1241 (306)		C4-O stretch + ring A breath
		1308 (91)	CH2 wag + NH_2 twist
		1325 (121)	phenol O-H bend + ring B breath
		1340 (74)	CH2 wag + CH bend
1330	1332 (83)		CH2 wag + C2'-C3' stretch + C5'-C6' stretch + ring B CH bends
1370	1394 (349)		carboxylic O-H bend
		1446 (455)	C4'-O stretch + ring B C-H bends
		1475 (91)	C4-O stretch + ring A C-H bends
1473	1472 (268)		C4-O stretch + ring A C-H bends
1527	1528 (310)		C4'-O stretch + C2'-C3' stretch + C5'-C6' stretch + ring B CH bends
1550	1565 (103)		C4-O stretch + C2-C3 stretch + C5-C6 stretch + ring A C-H bends
	1595 (52)		C2-C3 stretch + C5-C6 stretch
		1593 (59)	ring A breath + ring B breath
		1603 (225)	NH_2 sciss
		1650 (262)	CO_2 asym stretch + NH_2 sciss
1793	1788 (294)		CO_2 asym stretch + carboxylic O-H bend

[a] in cm^{-1}. [b] intensities in brackets in km mol^{-1}. Vibrations with intensities lower than 50 km mol^{-1} are not reported.
